# Loss of miR-192-5p initiates a hyperglycolysis and stemness positive feedback in hepatocellular carcinoma

**DOI:** 10.1186/s13046-020-01785-7

**Published:** 2020-11-30

**Authors:** Yuanzhuo Gu, Fubo Ji, Niya Liu, Yongzhi Zhao, Xiyang Wei, Shiyuan Hu, Wei Jia, Xin Wei Wang, Anuradha Budhu, Juling Ji, Bin Zhao, Stephanie Roessler, Xin Zheng, Junfang Ji

**Affiliations:** 1 MOE Key Laboratory of Biosystems Homeostasis & Protection and Zhejiang Provincial Key Laboratory for Cancer Molecular Cell Biology, Life Sciences Institute, Zhejiang University, 866 Yuhangtang Road, Hangzhou, 310058 Zhejiang Province China; 2 Hong Kong Baptist University, HongKong, China; 3 Liver Carcinogenesis Section, The Lab of Human Carcinogenesis, National Cancer Institute, Bethesda, MD 20892 USA; 4 Department of Pathology, Medical School of Nantong University, Nantong, 226019 Jiangsu Province China; 5 Institute of Pathology, University Hospital Heidelberg, 69120 Heidelberg, Germany; 6 EZKIT L.L.C, Honolulu, HI 96825 USA

**Keywords:** Hepatocellular carcinoma, Cancer stem cell, miR-192-5p, Glycolysis, C-Myc

## Abstract

**Background:**

Emerging studies revealed that cancer stem cells (CSCs) possessed peculiar metabolic properties, which however remained largely unknown in hepatocellular carcinoma (HCC). Genetic silencing of liver-abundant miR-192-5p was a key feature for multiple groups of CSC-positive HCCs. We thus aimed to investigate essential metabolic features of hepatic CSCs via using HCCs with miR-192-5p silencing as a model.

**Methods:**

Datasets from two independent HCC cohorts were used. Data integration analyses of miR-192-5p with metabolome and mRNA transcriptome data in HCC Cohort 1 were performed to investigate miR-192-5p related metabolic features, which was further validated in Cohort 2. Cellular and molecular assays were performed to examine whether and how miR-192-5p regulated the identified metabolic features. Co-culture systems consisting of HCC cells and LX2 (human hepatic stellate cell line) or THP1 (human monocyte cell line) were established to explore effects of the identified metabolic properties on stemness features of HCC cells via interacting with co-cultured non-tumor cells.

**Results:**

High levels of glycolysis-related metabolites and genes were present in HCCs with low miR-192-5p and CSC-positive HCCs in two independent HCC cohorts. miR-192-5p knockout cells displayed CSC features and miR-192-5p loss led to an enhanced glycolytic phenotype via upregulating three bona fide targets, GLUT1 and PFKFB3 (two glycolytic enzymes) and c-Myc (regulating glycolytic genes’ expression). Meanwhile, c-Myc suppressed miR-192-5p transcription, ensuring a low-miR-192-5p/high-c-Myc loop to maintain hyperglycolysis. Moreover, over-produced lactic acid from hyperglycolytic HCC cells stimulated the ERK phosphorylation of co-cultured LX2 and THP1 non-tumor cells partially via NDRG3 and MCT1, which in turn promoted cell malignancy and stemness of HCC cells. Consistently, HCC patients with low level of miR-192-5p in their tumor tissues and high level of NDRG3 or MCT1 in their non-tumor tissues had the shortest overall survival.

**Conclusions:**

In CSC-positive HCCs, miR-192-5p loss enhanced glycolysis and over produced lactate might further increase HCC malignant features via interacting with environmental non-tumor cells.

**Supplementary Information:**

The online version contains supplementary material available at 10.1186/s13046-020-01785-7.

## Background

Abnormal cancer metabolism is one of the 10 cancer hallmarks. Studies of cancer metabolism have revealed the important roles of metabolic reprogramming in cancer cells for proliferation, metastasis and drug resistance [1, 2]. In tumors, a small population of cancer cells exhibit a high capacity of self-renewal and tumor initiation in NOD/SCID mice, which are referred to as cancer stem cells (CSCs). Currently, CSCs are considered to be responsible for tumor initiation, therapy resistance as well as recurrence, and rising attentions have been placed on CSC metabolism [3–5]. Recent studies indicate that CSCs have different metabolic properties when compared to the tumor bulk and that metabolic plasticity is dependent on the intrinsic demand of nutrients as well as the surrounding environment [6–9]. CSCs exhibit distinct metabolic phenotypes that include low mitochondrial respiration, high glycolytic activity, and high fatty acid oxidation based on tumor types and CSC biomarkers as well as isolation methods [6, 10, 11]. Exploiting metabolic vulnerability of CSCs may provide new effective cancer therapies to diminish tumor recurrence and metastasis.

Primary liver cancer is the fourth lethal neoplasm worldwide, 90% of which are hepatocellular carcinoma (HCC) [12]. Hepatic CSCs are considered as one of the determining factors for HCC carcinogenesis and recurrence and multiple hepatic CSC biomarkers such as EpCAM, CD133, CD90, CD44, and CD24, have been used to enrich tumorigenic CSCs in both HCC cell lines and primary HCC tissues [13–19] . Although gene signatures and regulatory pathways of CSC^+^HCCs were thoroughly investigated, their metabolic properties remained largely unknown except for few recent studies. Three studies revealed a high glycolysis rate and a low oxygen consumption rate (OCR) in CD133^+^ hepatic CSCs [20], the importance of fatty acid oxidation in CD133^+^ CSCs [21], and increased lipid metabolites associated with stearoyl-CoA-desaturase in EpCAM^+^ HCCs [22]. Hence, it is essential to systematically investigate the key metabolic features shared among different CSC biomarker positive HCCs.

MiR-192-5p is the 2nd most abundant miRNA in the liver [23]. Its genetic silencing frequently occurs in many groups of hepatic CSC marker-positive HCCs such as EpCAM^+^, CD90^+^, CD133^+^, CD44^+^ and CD24^+^HCCs, as well as pluripotency marker-positive HCCs [23]. HCC cells with miR-192-5p loss display a highly invasive phenotype and essential CSC features partially mediated via the p53/miR-192-5p/PABPC4 pathway [23–25]. Thus, investigating key metabolic features in HCCs with miR-192-5p loss might allow us to explore the essential metabolic properties among multiple different groups of CSC^+^HCCs.

## Methods

### Omics dataset

**Fig. 1:**
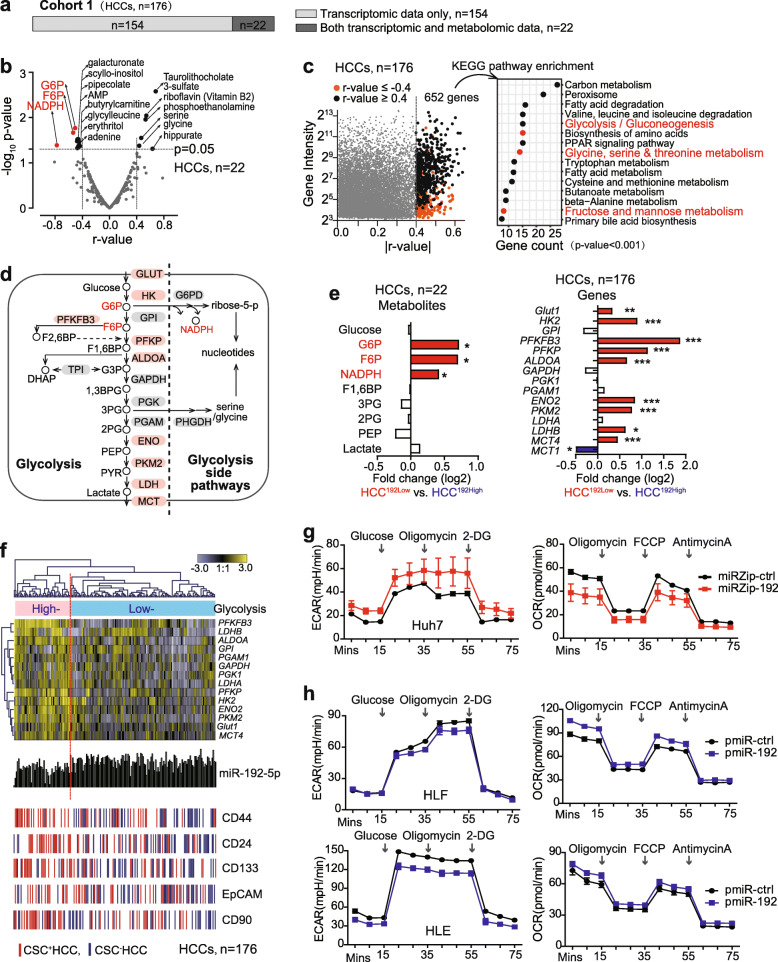
In HCCs, high expression of glycolysis-related metabolites and genes was associated with low miR-192-5p level. **a** Available omics dataset in Cohort 1 (*n* = 176). **b** Spearman correlation was performed between miR-192-5p and metabolome in 22 HCC cases of HCC Cohort 1. 17 metabolites were significantly correlated with miR-192-5p with |r-value| > 0.4 (*p* < 0.05). **c** 652 genes significantly correlated with miR-192-5p (Pearson correlation, |r-value| > 0.4) in Cohort 1 were used for KEGG pathway enrichment analysis. **d** Major metabolites and genes in glycolysis and glycolysis side pathways. **e** Log_2_ ratios of glycolysis-related metabolites and genes in HCC^192Low^ vs. HCC^192High^ HCCs of Cohort 1. **f** Hierarchical clustering analysis with glycolysis-related genes in Cohort 1. Red bar and green bar represent CSC biomarker positive and negative HCC cases, respectively. miR-192-5p expression levels were also shown. **g** ECAR and OCR measurement in Huh7 cells infected with lentivirus miRZip-ctrl or miRZip-192. **h** ECAR and OCR measurement in HLF and HLE cells infected with lentivirus pmiR-ctrl or pmiR-192

A total of five datasets from two HCC cohorts with 548 cases were used. In Cohort 1, there were a total of 176 Asian HCC cases, among which 22 cases had available metabolomics data and all cases had miRNA transcriptome (GSE6857) and mRNA transcriptome data (GSE14520) [22, 23, 26–29] (Fig. 1a). In Cohort 2, there were 372 HCC cases with different races. Their miRNA and mRNA sequencing data were downloaded from The Cancer Genome Atlas (TCGA) and available *TP53* mutation status and *MYC* amplification information in 240 HCC cases were collected from www.cbioportal.org/index.do.

### Cell lines and miR-192-5p knockout HCC cells

Human liver cancer cell lines including HLF, HLE, Huh7 and HepG2 cells; human embryonic kidney HEK293T cells; human hepatic stellate cell (HSC) line LX2 cells; human hepatocyte line HL7702 cells; and human leukemic monocyte cell line THP1 (with macrophage activation) were used in this study as described before [23, 30]. HLF, HLE and Huh7 were originally from Japanese Collection of Research Biosources Cell Bank (JCRB). HepG2, 293 T and THP1 were from American Type Culture Collection (ATCC), and LX2 and HL7702 were from Chinese Academy of Sciences (Shanghai, China). Cells were cultured in Dulbecco’s modified Eagle’s medium (DMEM) supplemented with 10% FBS and 100 U/mL penicillin–streptomycin, 1% L-glutamine. Cells were grown at 37 °C in a 5% CO2 incubator and regularly tested to be free of mycoplasma contamination.

MiR-192-5p knockout HCC cells were generated using the CRISPR/Cas9 system. Two single-guide RNAs (sgRNAs) were designed via an online web tool (http://crispr.mit.edu) to target the precursor mir-192. The synthesized sequences were cloned into pSpCas9(BB)-2A-Puro (PX459) vector, termed as PX459-sgMiR192. 48 h after PX459-sgMiR192 transfection, HCC cells underwent puromycin selection (1.5 μg/ml) for 5 days. Next, 1000 collected cells were seeded in a 10-cm dish and cultured for 10–12 days to select miR-192-5p knockout single cell clones. Positive clones were identified via PCR examination and sequencing validation. The sequences for guide RNAs and PCR primers are listed in Supplementary Table S1.

### Plasmids, siRNAs and cell treatments

Lentiviral constructs pre-hsa-miR-control (pmiR-control) and pre-hsa-miR-192-5p (pmiR-192) with GFP as well as miRzip-control and miRZip-192-5p (miRZip-192) were obtained from SBI Biosciences and stored in our laboratory. pmiR-control with RFP (pmiR-ctrl/RFP) and pmiR-192 with RFP (pmiR-192/RFP) were constructed by replacing GFP with Ds-Red between the HindIII and NotI sites. Lentiviruses were packaged with plasmids psPAX2 and pMD2.G (Addgene) in 293 T cells. For infection, 5 MOI of each lentivirus was used for all our studies.

pMiR-Report-control (Luc-Ctrl), pmiR-192 reporter (192pos reporter), and miRZip-192 reporter (Zip192 reporter) were constructed before [23]. The miR-192-5p binding regions in the 3’UTR or coding region of *GLUT1*, *PFKPB3*, and *MYC* were inserted into the monoclonal sites (HindIII/SpeI) of the pMiR-Report plasmid to generate the corresponding luciferase reporters. The pT3-EF1α-c-Myc vector was originally modified from pT3-EF1α for c-Myc overexpression [31]. pGL-miR-192-5p (pGL-192) plasmids were constructed by inserting different lengths of the miR-192 promoter region to the monoclonal site (KpnI/XhoI) of pGL4.20-basic (Promega).

*PFKFB3* siRNAs, *GLUT*1 siRNAs and negative control siRNA were purchased from RiboBio Co, Guangzhuo, China. *TP53* siRNAs, *MYC* siRNAs, *NDRG3* siRNAs, and *MCT1* siRNAs were purchased from GenePharma Co., Shanghai, China. Lipofectamine 2000 (Invitrogen) reagent and Rfect siRNA Transfection Reagent (BIO-TRAN) were used for the transfections of plasmids and siRNAs, respectively. The detailed information of all primers and sequences is listed in Supplementary Table S1.

Nutlin-3a (Selleck Chemicals) was dissolved in DMSO. When indicated, HCC cells were treated for 24 h with 10 μM of Nutlin-3a. 2-DG (Sigma-Aldrich) was dissolved in ddH_2_O and HCC cells were treated for 72 h with 5–10 μM of 2-DG.

### Extracellular acidification rate (ECAR) and oxygen consumption rate (OCR) assays

In the XF8 Extracellular Flux Analyzer (Seahorse Bioscience), the ECAR and OCR were measured using the Seahorse XF Glycolysis Stress Test Kit (Agilent Technologies) and Seahorse XF Cell Mito Stress Test Kit (Agilent Technologies), respectively. Experiments were performed according to the manufacturer’s instructions. For the ECAR measurement, cells (1 × 10^4^ cells/well) were plated in an XF8 cell-culture microplate for 12 h and the culture medium was then replaced with XF assay medium supplemented plus 2 mM L-glutamine. After one-hour incubation at 37 °C, the ECAR was measured by the sequential addition of glucose (10 mM), oligomycin (3.5 μM), and 2-deoxyglucose (2DG, 80 mM) in an XF8 flux analyzer. For the OCR measurement, cells (1 × 10^4^ cells/well) were plated in an XF8 cell-culture microplate for 12 h and then culture medium was replaced with XF assay medium supplemented with 10 mM D-glucose, 1 mM sodium pyruvate and 2 mM L-glutamine. The OCR was measured by sequential addition of oligomycin (2 μM), FCCP (0.5 μM), and antimycin A (1 μM). Data were analyzed by Seahorse XF Wave software. The results were normalized to the cell number.

### Co-culture system

Two types of co-culture systems were used. In the first, HCC cells infected with corresponding RFP or GFP lentivirus were co-cultured with HL7702 or LX2 cells in the same dish. In this system, cells that displayed red or green under a fluorescence microscope or flow cytometry were regarded as HCC cells. The second co-culture system was based on a chamber system using Polyester (PET) Membrane Tissue Culture Plate Insert with 0.4 μm pores (JET biofil) according to the manufacturer’s protocol. Generally, LX2 or HL7702 were placed in the bottom layer of a 6-well plate while HCC cells were placed in the cell inserts of this 6-well plate. In addition, HCC cells were also co-cultured with THP1, a suspension cell line.

### RNA isolation, quantitative real-time PCR, and Western blot

Total RNA was isolated using TRIzol (Invitrogen) following the manufacturer’s instructions, and 1 μg of total RNA was reverse transcribed into cDNA using PrimeScript™ RT Reagent Kit with gDNA Eraser (TaKaRa). Quantitative reverse transcription polymerase chain reaction (qRT-PCR) was performed with TB Green™ Premix EX Taq™ II (Tli RNaseH Plus) (TaKaRa). The expression of mature miRNAs was measured using TaqMan MiRNA Assays as described previously [28, 32, 33]. RNU6B was used as the reference gene for miRNAs, while 18S was the reference for measured mRNAs. Primers for *TACSTD1, CD133, CD90, CD44, CD24,* and *UGT2B7* were used as before [23]*.* Primer sequences for *GLUT1*, *HK2, PFKP, PFKFB3, ALDOA, ENO2, PKM2, LDHA, MCT4, MCT1, NDRG3* and *CYP1A2* are listed in Supplementary Table S1. The experiments were performed in triplicate.

For the Western blot assay, cells were lysed and processed as previously described [28, 33]. The membranes with transferred protein extracts were incubated with the indicated primary antibodies and secondary antibodies conjugated to horseradish peroxidase for enhanced chemiluminescence detection of the signals (Amersham, Arlington Height, IL). The detailed information of all antibodies is listed in Supplementary Table S2.

### Non-targeted metabolomics study

The non-targeted metabolomics was performed by Metabo-Profile Biotechnology (Shanghai) Co., Ltd. Cell lysates and cell culture medium were collected from cells and used for metabolomics analysis. Metabolites were identified and quantified from gas chromatography/time-of-flight mass spectrometry (GC/TOF-MS) data, which was performed in the workflow of ADAP-GC 2.0. Each experiment was performed in triplicate.

### 2-NBDG uptake, glucose measurement and lactate measurement

Glucose uptake of HCC cells was quantified by flow cytometry using the fluorescent D-glucose derivate 2-(N-(7-nitrobenz-2-oxa-1,3-diazol-4-yl)amino)-2-deoxy-D-glucose (2-NBDG). Briefly, the corresponding cells were cultured with 2-NBDG at 37 °C for 1 h. The uptake of 2-NBDG was measured by flow cytometry. A glucose measurement kit (Shanghai Rongsheng Biotech Co., Ltd.) was used to measure the glucose concentration in cell culture medium. Extracellular lactate production was measured using a lactate assay kit (Nanjing Jiancheng Bioengineering Institute). All these assays were performed according to the manufacturer’s protocols.

### Sphere formation assay and flow cytometry analysis

Single-cell suspensions of 1000 cells were seeded in 6-well Ultra-Low Attachment Microplates (Corning, Corning, NY) for spheroid assays. The number of spheroids was measured 12 days after seeding.

For flow cytometry analysis, cultured cells were trypsinized, washed, and resuspended in phosphate-buffered saline plus 0.5% bovine serum albumin. They were incubated with Allophycocyanin (APC)-conjugated antibodies on ice for 20 mins in the dark. Data were collected with a FACS Calibur flow cytometer (BD Biosciences) and analyzed using FlowJo software (Tree Star). The detailed information for all antibodies is listed in Supplementary Table S2.

### Luciferase reporter assay

The pMiR-Report plasmids (with or without mature miR-192-5p binding sites) were transfected together with pRL-CMV vector containing Renilla luciferase. The Firefly and Renilla luciferase activities were measured 24 h after transfection using Dual-Luciferase Reporter Assay (Promega, CA) with a PerkinElmer luminometer. Each experiment was performed in triplicate and repeated at least three times.

To examine the promoter activity of miR-192-5p, HCC cells (24-well plate) were transfected with 300 ng pT3-EF1α vector or pT3-EF1α-cMyc vector, or 50 nM siRNAs (negative control or *MYC* siRNAs) on the first day and then transfected with 300 ng pGL-192 promoter constructs and 10 ng pRL-CMV on the next day. The Firefly and Renilla luciferase activities were then measured 24 h after transfection.

### Statistics

Spearman’s rank correlation was performed to identify metabolites associated to miR-192-5p. Student’s t-test and Mann-Whitney rank test were used for statistical analysis of comparative data between groups. Two-way ANOVA was used to compare the glucose consumption, lactate production and cell viability of HCC cells at different time points. Hierarchical clustering analysis was performed by GENESIS software version 1.7.6 developed by Alexander Sturn (IBMT-TUG, Graz, Austria). Pearson correlation was used to identify genes correlated with miR-192-5p. Kaplan–Meier survival analysis was used to compare patient survival based on prediction results using GraphPad Prism V7.0 (San Diego, CA), and the *p*-value was generated by the Cox–Mantel log-rank test. Gene set enrichment analysis (GSEA) in the Molecular Signatures Database was performed using GSEA V3.0. All *p*-values were 2-sided. A *p*-value of ≤0.05 was regarded as statistically significant.

## Results

### Glycolysis-related metabolites and genes were highly expressed in HCC cases with low miR-192-5p expression

We first investigated the metabolic features in HCCs with low miR-192-5p levels using HCC Cohort 1 with available metabolome and transcriptome data (Fig. 1a). We performed an integration analysis of miR-192-5p with the global metabolome in tumor tissues among 22 HCC patients, and found that 17 metabolites were significantly correlated with miR-192-5p (|r-value| > 0.4, Fig. 1b, Supplementary Table S3). Among them, 7 metabolites presented |r-value| > 0.5 and three of them were glycolysis-related metabolites, i.e., Glucose-6-Phosphate (G6P), Fructose-6-Phosphate (F6P), and Nicotinamide Adenine Dinucleotide Phosphate (NADPH). Meanwhile, 652 genes were significantly correlated with miR-192-5p with |r-value| > 0.4, revealed by an integration analysis of miR-192-5p with mRNA transcriptome in tumor tissues among 176 HCC patients (Fig. 1c). Kyoto Encyclopedia of Genes and Genomes (KEGG) pathway analysis using these genes revealed 13 enriched metabolic features (*p* < 0.001), three of which were associated with glycolysis and glycolysis-related pathways. These results suggest an altered glycolytic feature in HCC cases with low miR-192-5p expression.

Available glycolysis-related metabolites and genes in our profiling data (Fig. 1d) were then compared between HCCs with high miR-192-5p levels (termed HCC^192High^) and HCCs with low miR-192-5p levels (termed HCC^192Low^), based on a miR-192-5p median cut-off in HCC tumors. Levels of G6P, F6P, and NADPH were significantly higher in tumors from HCC^192Low^ patients than HCC^192High^ patients, while no difference was found in their non-tumor tissues (Fig. 1e, Supplementary Fig. S1a). Consistently, many genes coding for key glycolytic enzymes such as *GLUT1, HK2, PFKFB3, PFKP, and PKM2* were significantly upregulated in HCC^192Low^ tumors compared to HCC^192High^ tumors (Fig. 1e) but showed negligible alteration in their non-tumor tissues (Supplementary Fig. S1b). *MCT1* was used as a negative control due to its main role in lactate import, but not in glycolysis [34, 35]. These results demonstrate a hyperglycolytic metabolic feature in HCC cases with low miR-192-5p level.

We further investigated the hyperglycolytic feature in CSC^+^ HCC cases, i.e., cases with the top quartile expression of CSC markers as previously defined [23]. Hierarchical clustering analysis with glycolytic genes in Cohort 1 revealed two HCC subgroups with different expression levels of glycolytic genes. Consistently, in HCC subgroup with high expression levels of glycolysis-related genes, miR-192-5p level was low while various groups of CSC^+^ HCC cases were enriched (Fig. 1f). Statistical analysis also showed that glycolytic genes were expressed at significantly higher levels in various groups of CSC^+^ HCCs than in CSC^−^ HCCs, but no difference was observed in the comparisons of their non-tumor tissues (Supplementary Fig. S1c-d). Comparable data were observed in Cohort 2 with 372 HCC patients (Supplementary Fig. S1e). Together, these data indicated that the hyperglycolytic feature was present in various groups of CSC^+^HCCs with low level of miR-192-5p.

### HCC cells with miR-192-5p loss were hyperglycolytic

We next investigated the role of miR-192-5p in regulating glycolysis. In Huh7, glycolytic genes with significant differential expression between HCC^192High^ and HCC^192Low^ patients were examined and eight out of nine genes showed a significant up-regulation after suppressing miR-192-5p (Supplementary Fig. S2a). Meanwhile, Huh7 cells with suppressed miR-192-5p exhibited a distinctly increased ECAR and a reduced OCR (Fig. 1g). The extracellular acid produced by cells is derived from lactate produced by glycolysis and CO_2_ produced during respiration. OCR mainly represents mitochondrial respiration. Therefore, the increased ECAR in Huh7 cells with suppressed miR-192-5p was mainly due to lactate produced from glycolysis but not CO_2_ from mitochondrial respiration. Consistently, over-expressed miR-192-5p in HLF and HLE cells lowered the extracellular acid production from glycolysis as shown by a reduced ECAR but an increased OCR (Fig. 1h). Most examined glycolytic genes were significantly reduced by miR-192-5p overexpression in both HLF and HLE cells (Supplementary Fig. S2b). These results demonstrate an important role of miR-192-5p in modulating a Warburg-like effect in HCC cells.

**Fig. 2:**
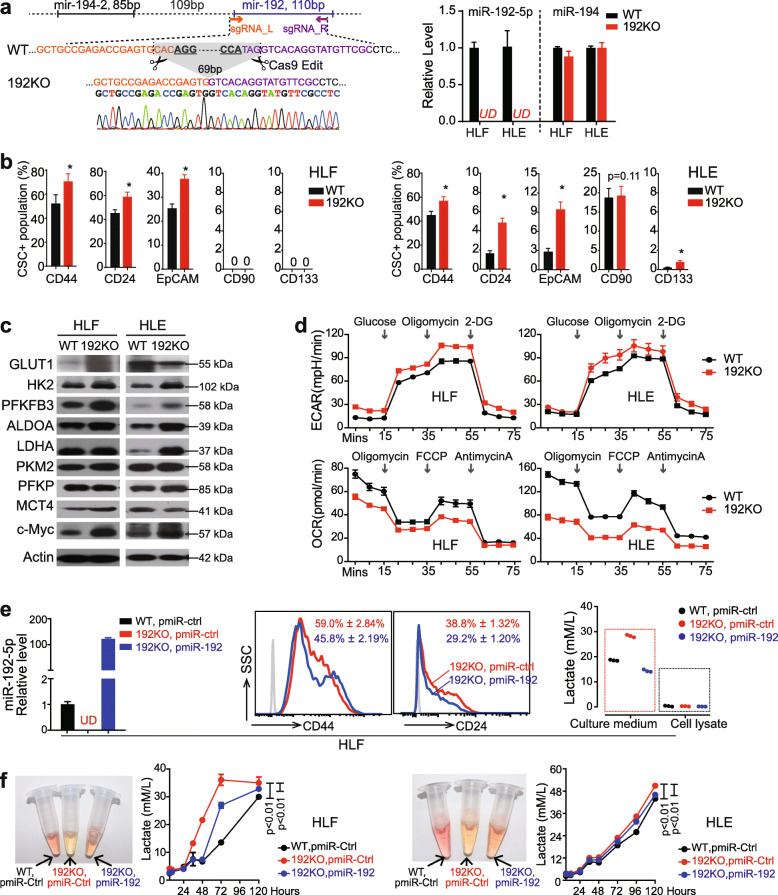
MiR-192-5p knockout human HCC cell lines presented the increased CSC features and were highly glycolytic. **a** Schematic structure of miR-192-5p wild-type (WT) and knockout (KO) by CRISPR/Cas9 system. The knockout of miR-192-5p DNA fragment was assessed by Sanger sequencing. Levels of miR-192-5p and miR-194 was measured by RT-qPCR. **b** Quantitative data of flow cytometry analysis using APC-conjugated antibodies against different CSC biomarkers in 192KO lines and WT lines from HLF and HLE cells. **c** Western blot of glycolytic enzymes using 192KO lines and WT lines from HLF and HLE cells. **d** ECAR and OCR were measured in 192KO lines and WT lines from HLF and HLE cells. **e** RT-qPCR analysis of miR-192-5p, flow cytometry analysis of CD24^+^ and CD44^+^ populations, and lactate measurement using Lactate assay kit in HLF cells with different levels of miR-192-5p. **f** Measurement of lactate in cultured medium from HLF and HLE cells at different time points. Representative images for color change of the corresponding cultured medium at 72 h after seeding are shown

To better elucidate the role of miR-192-5p in regulating glycolysis, we established miR-192-5p knockout (termed 192KO) clones from two human HCC cell lines HLF and HLE. A 69 bp DNA fragment was deleted in the 192KO mixture clones as well as selected single 192KO clones (Supplementary Fig. S2c-d). One 192KO single clone from each HCC cell lines was used and miR-192-5p expression was undetectable in HLE-192KO and HLF-192KO cells (Fig. 2a). As a control, the expression of miR-194, a nearby miRNA of miR-192-5p, was not affected. As expected, HLF-192KO cells displayed significantly increased CSC features, such as increased populations of CD44^+^, CD24^+^ and EpCAM^+^ CSCs (Fig. 2b, Supplementary Fig. S2e); increased mRNA levels of multiple CSC biomarkers and reduced expression of a differentiation-related gene *CYP1A2* (Supplementary Fig. S2f); and enlarged and more spheroid formation (Supplementary Fig. S2g). HLE-192KO cells displayed increased CSC features at a moderate level (Fig. 2b, Supplementary Fig. S2e-f). Consistently, these two 192KO lines also showed the hyperglycolytic features. As shown in Fig. 2c, five glycolytic enzymes, i.e., GLUT1, HK2, PFKFB3, ALDOA, and PKM2, as well as c-Myc presented higher levels in 192KO cells than in wild-type cells. 192KO lines also exhibited increased ECARs but decreased OCRs (Fig. 2d), indicating that miR-192-5p loss largely increased the glycolysis-related extracellular acidification. Consistent data were also noticed in other 192KO clones of both HLF and HLE HCC cell lines (Supplementary Fig. S3a-b).

Furthermore, overexpressed miR-192-5p in HLF-192KO cells significantly reduced CSC features and lactate accumulation in the culture medium (Fig. 2e). As a control, the intracellular lactate remained unchanged. Meanwhile, over culturing time, lactate gradually accumulated in the medium and was significantly higher in both HLF-192KO and HLE-192KO cells compared to their corresponding wild-type cells, which could be lowered by overexpressed miR-192-5p (Fig. 2f). A lower pH value was also observed in 192KO cells indicated by the orange/yellow medium vs. the pink medium of wild-type cells at 72 h after seeding. Comparable data on lactate production were seen in other HLF and HLE 192KO clones (Supplementary Fig. S3c). Consistent data were also obtained through the detection of lactate using non-targeted metabolomics in HLF cells with different expression of miR-192-5p in both internal cells and culture medium (Supplementary Fig. S3d). Together, miR-192-5p loss in HCC cells led to a hyperglycolytic phenotype.

### HCC cells with miR-192-5p loss had high glucose consumption

**Fig. 3:**
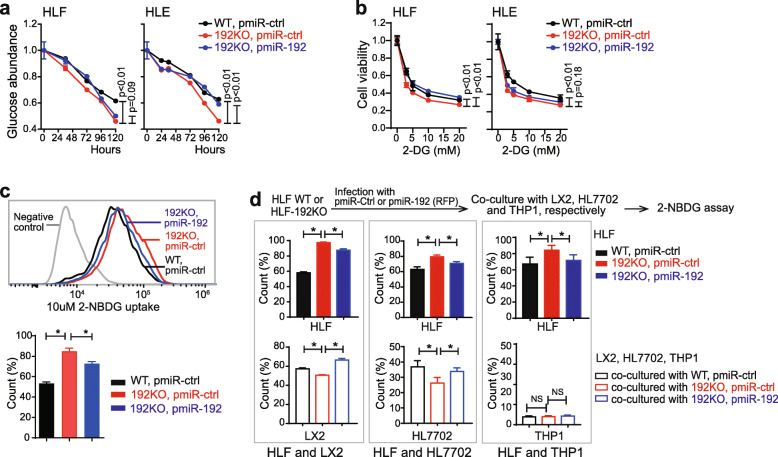
MiR-192-5p KO cells consumed more glucose from the environment. **a** Glucose concentration in culture medium from WT and 192KO lines of HLE and HLF cells infected with lentivirus pmiR-Ctrl or pmiR-192. **b** Cell viability of WT and 192KO lines for HLE and HLF cells infected with lentivirus pmiR-Ctrl or pmiR-192 and exposed to different doses of 2-DG. **c** 2-NBDG uptake by flow cytometry analysis of HLF-WT and -192KO cells infected with lentivirus pmiR-Ctrl (RFP) or pmiR-192 (RFP). **d** 2-NBDG uptake for all cells in co-culture system. HLF cells were infected with lentivirus pmiR-Ctrl (RFP) or pmiR-192 (RFP) and co-cultured with LX2, HL7702 or THP1 cells. Two-way ANOVA analysis was performed for (**a**, **b**) and student t-test was performed for (**c**, **d**). *, *p* < 0.05

We further examined glucose consumption among HCC cells with different levels of miR-192-5p as well as between HCC cells and their co-cultured non-tumor cells. As shown in Fig. 3a, both HLF-192KO and HLE-192KO cells exhibited significantly higher glucose consumption than HCC cells overexpressing miR-192-5p. In Huh7 cells, suppressing miR-192-5p increased their glucose usage (Supplementary Fig. S4a). Consistently, 192KO cells were more sensitive after exposure to 2-DG, a glucose analog, as shown by the significantly reduced cell viability compared to cells with miR-192-5p expression (Fig. 3b). Comparable data were obtained in HuH7 cells (Supplementary Fig. S4b).

In co-culture systems of HCC cells with LX2, HL7702, and THP1, we further compared their glucose uptake via 2-NBDG uptake assay. HLF HCC cells infected with pmiR-ctrl/RFP or pmiR-192/RFP lentiviruses were used, and red fluorescent labeling efficiency was nearly 100% (Supplementary Fig. S4c). In this system, with or without co-culturing with other cells, HLF-192KO cells consistently showed higher 2-NBDG uptake than wild-type cells (Fig. 3c-d). In contrast, LX2 and HL7702 in co-culture with HLF-192KO cells exhibited lower 2-NBDG uptake compared to those in co-culture with HLF-WT cells. Moreover, forced-expression of miR-192-5p in HLF-192KO cells reduced the 2-NBDG uptake in HLF cells but increased 2-NBDG uptake in LX2 and HL7702 cells in the co-culture system. The alteration of 2-NBDG uptake was not observed in THP1 from our co-culture system (Fig. 3d, Supplementary Fig. S4d). Similar data were seen in HLE cells as well as in HLE cells co-cultured with LX2 and HL7702 (Supplementary Fig. S4e-f). These results demonstrate that HCC cells with loss of miR-192-5p actively utilize glucose from their environment to ensure a hyperglycolysis status.

### GLUT1, PFKFB3 and c-Myc were miR-192-5p bona fide targets and contributed to glycolytic and stemness features of HCC cells

**Fig. 4:**
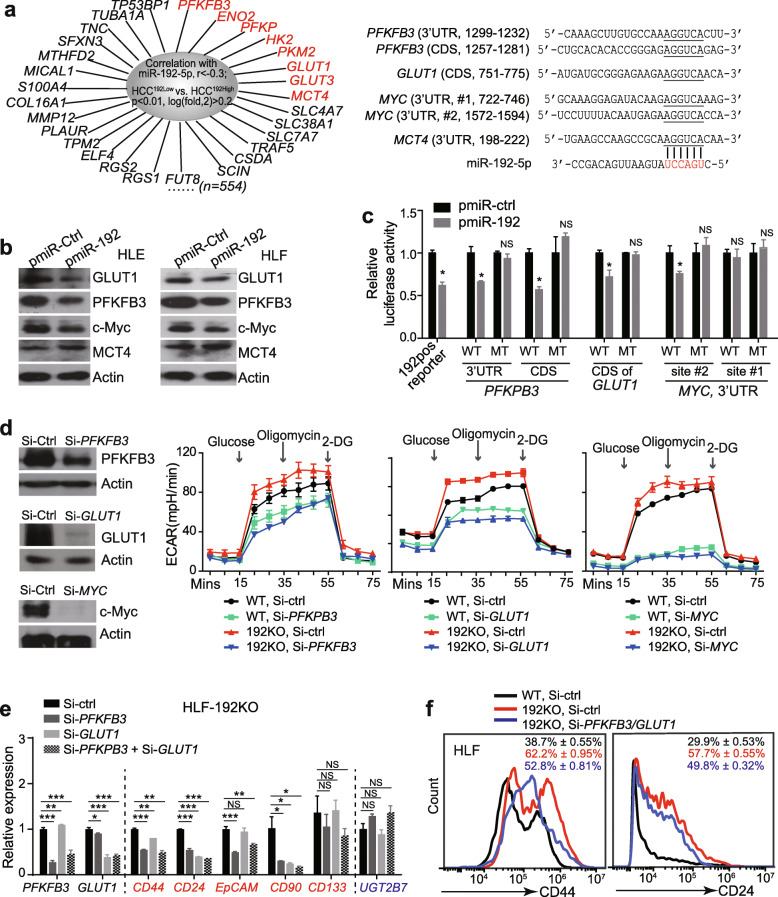
c-Myc as well as two glycolytic enzymes were miR-192-5p targets. **a** Genes negatively correlated with miR-192-5p (*r* < − 0.3) and significantly altered in HCC^192Low^ vs. HCC^192High^ (Left panel) and predicted miR-192-5p binding sites in 3′-UTR or coding regions (CDS) of human *PFKFB3*, *GLUT1*, *MYC* and *MCT4* (Right panel). **b** Western blotting of PFKFB3, GLUT1, c-Myc and MCT4 in HLE and HLF cells infected with lentivirus pmiR-Ctrl or pmiR-192. **c** Luciferase activities were measured using reporters with WT or MT miR-192-5p binding sites of three genes in the 3′-UTR or CDS of luciferase vector. 192pos reporter was used as the positive control. **d** ECAR was measured in HLF-WT and HLF-192KO cells transfected with 50 nM si-Ctrl, si-*PFKFB3*, si-*GLUT1*, and si-*MYC* respectively. **e** RT-PCR analysis of five CSC surface markers in HLF-WT and HLF-192KO cells infected with si-Ctrl, si-*PFKFB3*, si-*GLUT1*, as well as the combination of si-*PFKFB3* and si-*GLUT1*. *UGT2B7* was measured as a negative control. **f** Flow cytometry analysis of CD24^+^ and CD44^+^ populations in HLF-WT cells transfected with si-Ctrl, HLF-192KO cells transfected with si-Ctrl, and HLF-192KO cells transfected with the combined si-*PFKFB3* and si-*GLUT1*. Student t-test was performed for (c.e.). *, *p* < 0.05

To investigate the target genes of miR-192-5p in regulating glycolysis flow, we assessed genes negatively correlated with miR-192-5p in 176 HCC cases (r < − 0.3) and significantly up-regulated in HCC^192Low^ tumors versus HCC^192High^ tumors (log_2_fold > 0.2, *p* < 0.01). Among these 554 genes, two main groups were observed. One group contained genes related to cell migration as we previously reported [23]. The other group included eight glycolysis-related genes (Fig. 4a) and three of them (*GLUT1, HK2*, and *PKM2*) were reported targets of c-Myc, an important regulator of glycolysis [36, 37].

Next, using TargetScan and manual miRNA target prediction, we found that four of these glycolytic genes (*PFKFB3, GLUT1, MCT4*, and *MYC*) contained miR-192-5p binding sites in their 3’UTR and/or coding regions (Fig. 4a). In HLF and HLE cells, miR-192-5p overexpression reduced the protein levels of PFKFB3, GLUT1, and c-Myc, but not that of MCT4 (Fig. 4b). Further, the predicted miR-192-5p binding regions of these three genes were cloned into a luciferase reporter and forced expression of miR-192-5p reduced the luciferase activities when the wild-type sequences for *PFKFB3* and *GLUT1* as well as the #2 binding site of *MYC* were present (Fig. 4c). These effects were significantly reduced when the corresponding miR-192-5p binding sites were mutated. Moreover, silencing PFKFB3, GLUT1, or c-Myc with 2 siRNAs for each gene reduced ECAR in both HLF-WT and HLF-192KO cells (Fig. 4d, Supplementary Fig. S5a). In HLF-192KO cells, silencing PFKFB3, GLUT1 or c-Myc notably reduced the ECAR rate to a level similar to that of HLF-WT cells with silencing of these genes. Comparable data were also noticed in HCC patients from Cohorts 1 and 2. *PFKFB3*, *GLUT1*, and *MYC* presented higher levels in HCC^192Low^ tumors compared to HCC^192High^ tumors (Supplementary Fig. S5b-c). These results indicate that PFKFB3, GLUT1, and c-Myc are miR-192-5p targets and are involved in the hyperglycolysis caused by miR-192-5p loss.

PFKFB3, GLUT1, and c-Myc were reported to maintain stemness features in cancer at certain levels [38–41]. Consistently, si-*PFKFB3,* si-*GLUT1* or double knockdown led to reduced levels of four CSC biomarkers, i.e., *CD44*, *CD24*, *EpCAM* and *CD90*, as determined by RT-qPCR (Fig. 4e). Flow cytometry analysis also showed that si-*PFKFB3* and *GLUT1* reduced the populations of CD44^+^ and CD24^+^ CSCs (Fig. 4f). Meanwhile, si-*MYC* seemed to only reduce CD44^+^ CSCs moderately, but not the CD24^+^ CSC population (Supplementary Fig. S5d). Together, these data demonstrate that three glycolytic regulators, PFKFB3, GLUT1 and c-Myc were bona fide targets of miR-192-5p, and they contributed to both hyper-glycolysis and CSC features of HCCs caused by loss of miR-192-5p.

### C-Myc suppressed miR-192-5p transcription, ensuring a positive feedback of high c-Myc/low miR-192-5p in hyperglycolytic CSC^+^HCCs

**Fig. 5:**
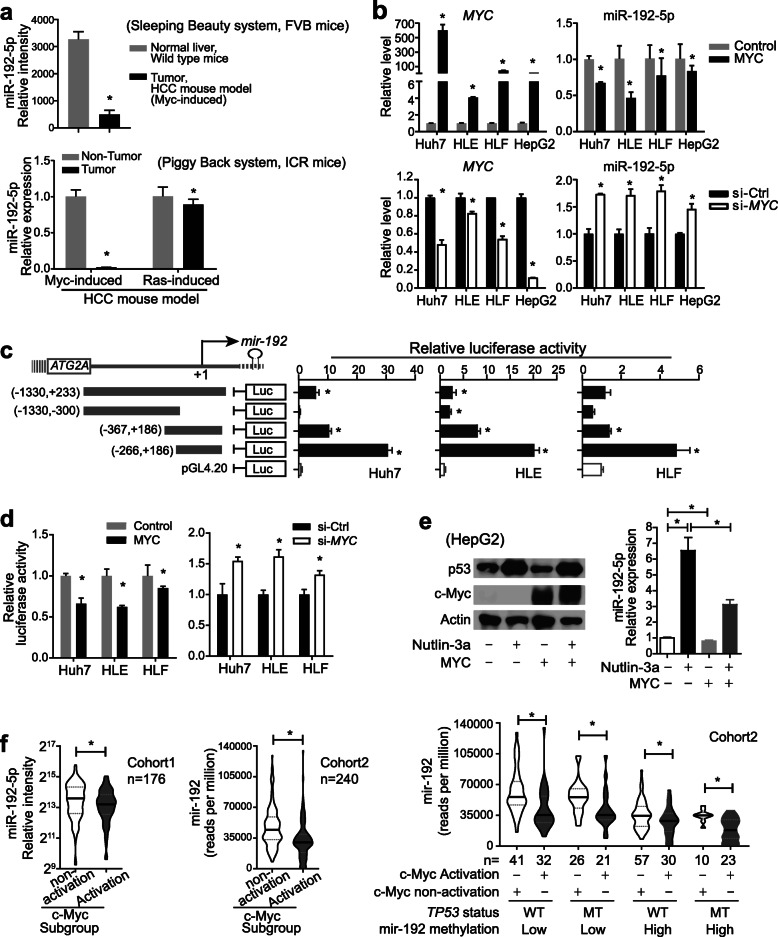
C-Myc activation reduced miR-192-5p expression in HCC. **a** RT-qPCR analysis of miR-192-5p in murine oncogene-induced HCC samples and the corresponding liver controls. RNAs from 3 to 5 mice for each group were used. **b** RT-qPCR analysis of *MYC* and miR-192-5p in HCC cell lines (Huh7, HLE, HLF and HepG2) transfected with Ctrl or *MYC* overexpressing plasmids, as well as with Ctrl or *MYC* siRNAs, respectively. **c** Dual-luciferase assays for different miR-192-5p promoter fragments in HLE, HLF and HuH7 cells. **d** Dual-luciferase assay using miR-192-5p promoter (− 266 nt, + 186 nt) in HLE, HLF and HuH7 cells transfected with Ctrl or *MYC* overexpressing plasmids, and with Ctrl or *MYC* siRNAs, respectively. **e** Western blotting analysis for p53 and c-Myc, and RT-qPCR analysis for miR-192-5p in HepG2 treated with Nutlin-3a (10uM) and/or transfected with *MYC*. **f** miR-192-5p expression levels were shown in c-Myc activation group and c-Myc non-activation group defined in Supplementary Fig. S6c-d in two cohorts. In Cohort 2, mir-192 expression levels were further shown in subgroups with different status of p53 mutation, mir-192 promoter methylation and c-Myc activation. Student t-test was performed. *, *p* < 0.05

In our previous miRNA profiles of tumors and non-tumors from a hydrodynamic injection HCC FVB mouse model [31], miR-192-5p expression was significantly reduced in c-Myc-induced HCCs (Fig. 5a). Further, in a hydrodynamic injection HCC ICR mouse model, miR-192-5p level was also reduced > 100 times in c-Myc induced HCCs but was not much in Ras-induced HCCs when compared to corresponding non-HCC liver tissues. In four different HCC cell lines, si-*MYC* led to an increased expression of miR-192-5p, while forced expression of c-Myc reduced the level of miR-192-5p (Fig. 5b). These data indicate that c-Myc might regulate miR-192-5p transcription.

Consistently, among four different lengths of miR-192-5p promoter regions, the − 266 nt to + 186 nt region showed the strongest promoter activity (Fig. 5c) and the miR-192-5p promoter activity (− 266 nt to + 186 nt) was reduced by exogenous c-Myc, while enhanced by si-*MYC* (Fig. 5d). Wild-type p53 could bind to the miR-192-5p promoter region and induce its expression [23, 42]. Consistently, in HepG2 cells with wild-type *TP53,* the expression of miR-192-5p was induced by p53 via exposure to Nutlin-3a (an MDM2 antagonist to stabilize p53) and reduced by silencing of p53 (Fig. 5e, Supplementary Fig. S6a-b). Over-expressed c-Myc significantly suppressed miR-192-5p expression in HCC cells with either activated p53 or silenced p53, indicating that c-Myc-mediated miR-192-5p down-regulation was independent on p53.

Comparable data were noticed in HCC patients. In both HCC cohorts, hierarchical clustering analysis revealed two subgroups with distinct c-Myc activation status based on 76 c-Myc target genes from the online Human MYC Targets Profiler (Supplementary Fig. S6c-d). In Cohort 1, miR-192-5p expression in the c-Myc activation subgroup was significantly lower than that in c-Myc non-activation subgroup (Fig. 5f). In Cohort 2, miR-192-5p expression was always significantly lower in each c-Myc activation subgroup than in the corresponding non-activation subgroup, which was independent of *TP53* mutation and mir-192 promoter methylation (Fig. 5 f-g). In addition, different groups of CSC^+^HCCs consistently presented a low level of miR-192-5p, a high level of c-Myc activation and high frequency of *MYC* amplification (Supplementary Fig. S7). Together, c-Myc suppressed miR-192-5p transcription, which led to a positive feedback of high c-Myc/low miR-192-5p in CSC^+^HCC cells with glycolytic feature.

### Overproduced lactate from CSC^+^HCCs activated the ERK pathway in environmental non-tumor cells, and this effect further increased HCC cell stemness and malignancy features

**Fig. 6:**
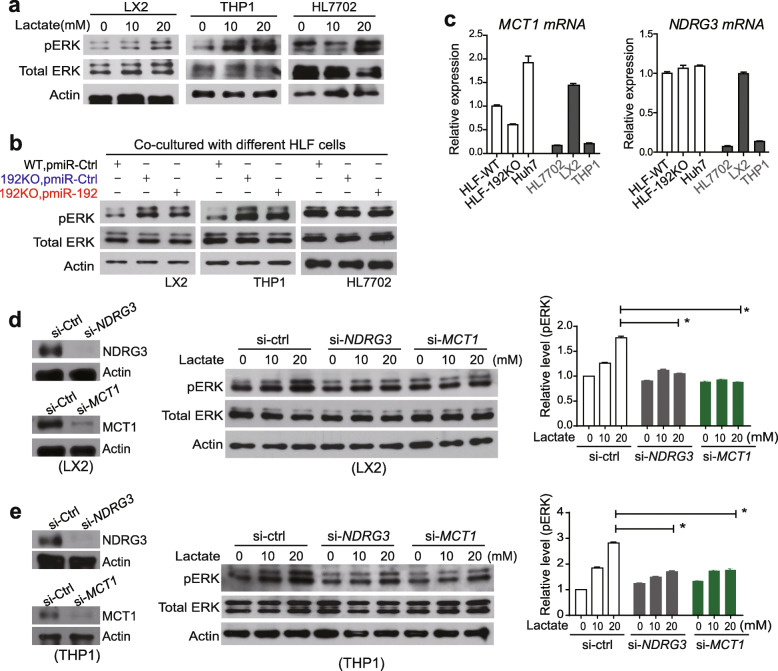
Overproduced lactate from HCC cells with miR-192-5p loss activated ERK pathway in environmental non-tumor cells. **a** Western blotting analysis for pERK and total ERK in LX2, THP1 and HL7702 cells treated with 0, 10, 20 mM lactate. **b** Western blotting analysis for pERK and total ERK in LX2, THP1 and HL7702 cells upon co-culture with HLF-WT or -192KO cells infected with lentivirus pmiR-Ctrl or pmiR-192 in a co-culture chamber system. **c** RT-qPCR analysis of *MCT1* and *NDRG3* in different HCC and non-HCC cell lines. **d** Western blot analysis of pERK and total ERK in LX2 cells transfected with si-*NDRG3* or si-*MCT1* and treated with different doses of Lactate. **e** Western blot analysis of pERK and total ERK in THP1 cells transfected with si-*NDRG3* or si-*MCT1* and treated with different doses of lactate. Student t-test was performed. *, *p* < 0.05

As the end product of glycolysis, the continuously produced lactate in hyperglycolytic miR-192-5p-loss HCC cells might affect their environment and contribute to HCC malignancy. The transport of lactate across the plasma membrane is mainly catalyzed by MCT1 and MCT4, with MCT1 typically involved in the import while MCT4 in export of lactate [34, 35]. In HCC patients, the expression ratio of *MCT1* vs. *MCT4* showed no difference between tumor and non-tumor tissues of Cohort 1 but was significantly higher in non-tumor tissues than tumor tissues of Cohort 2 (Supplementary Fig. S8a), indicating the possibility of lactate uptake by environmental non-tumor cells. Lactate could also stabilize NDRG3, which in turn activated the ERK pathway to promote cell malignancy [43]. Consistently, lactate treatment stimulated ERK phosphorylation noticeably in non-tumor cells of HCC microenvironment, i.e., LX2, THP1 and HL7702 cells (Fig. 6a).

In a chamber co-culture system, pERK level was increased in LX2 and THP1 cells when co-cultured with HLF-192KO cells compared to when co-cultured with HLF-WT (Fig. 6b). Moreover, pERK was further reduced in LX2 and THP1 when exposed to HLF-192KO cells with miR-192-5p overexpression (Fig. 6b). Similar results were observed from LX2 and THP1 cells co-cultured with HLE cells (Supplementary Fig. S8b). However, pERK was unaltered in HL7702 cells in this co-culture system. Thus, HCC cells with miR-192-5p loss could actively affect certain non-tumor cells via increased production of lactate.

We have also found that lactate-induced pERK in environmental non-tumor cells partially relied on NDRG3 and MCT1 (Fig. 6 c-e). LX2 and THP1 cells expressed relatively high levels of *MCT1* and *NDRG3* (Fig. 6c). Silencing NDRG3 or MCT1 in LX2 and THP1 cells reduced the level of lactate-induced pERK (Fig. 6d-e). Low expression levels of MCT1 and NDRG3 in HL7702 were consistent with its minor response to lactate (data not shown).

**Fig. 7:**
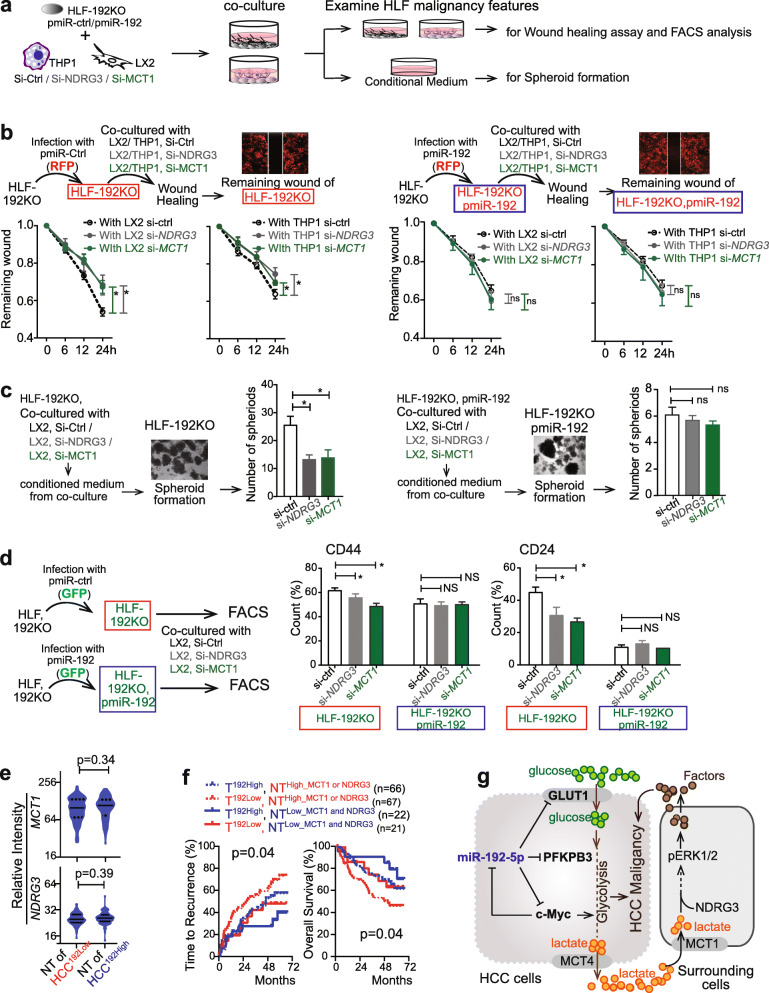
Silencing *MCT1* and *NDRG3* in LX2 or THP1 reduced malignancy and stemness features of co-cultured HCC cells. **a** The schematic diagram of examining malignancy features of HCC cells under a co-culture condition. **b** For wound healing assay, scratches were generated in a confluent monolayer HLF cells infected with pmiR-ctrl/RFP or pmiR-192/RFP which were co-cultured with LX2 or THP1 pre-transfected with si-*NDRG3* or si-*MCT1*. **c** HLF-192KO infected with pmiR-ctrl/GFP or pmiR-192/GFP were used for spheroid formation assay. Conditioned medium was used for this assay and collected from corresponding HLF cells co-cultured with LX2 pre-transfected with si-*NDRG3* or si-*MCT1.* **d** Quantitative data of flow cytometry analysis of CD24^+^ and CD44^+^ populations in HLF-192KO cells or HLF-192KO infected with pmiR-192/GFP cells co-cultured with LX2 pre-transfected with si-*NDRG3* or si-*MCT1*. **e** Relative levels of *MCT1* and *NDRG3* in non-tumor samples from patients with different levels of miR-192-5p in their tumors. **f** Kaplan–Meier curves of overall survival and time to recurrence in Cohort 1 according to miR-192-5p level in tumors as well as *MCT1* or *NDRG3* levels in non-tumors. **g** The schematic model of the miR-192-5p regulatory pathway in glycolysis and hepatic CSC features. Student t-test was used for (**c**, **d** and **e**). *, *p* < 0.05

We then explored the effects of an altered lactate/MCT1/NDRG3/pERK axis in LX2 or THP1 cells on the malignancy features in HCC cells (Fig. 7a). HLF-192KO/RFP cells were co-cultured with LX2 cells pre-transfected with si-Ctrl, or si-*NDRG3*, or si-*MCT1* (termed LX2^si-Ctrl^, LX2^si-*NDRG3*^, and LX2^si-*MCT1*^*,* respectively). Wound-healing assay of red-fluorescence HCC cells showed that cell migration of HLF-192KO cells was slower upon co-culture with LX2^si-*NDRG3*^ or LX2^si-*MCT1*^ than with LX2^si-Ctrl^ (Fig. 7b). Consistent data were observed in HLF-192KO cells co-cultured with THP1 (Fig. 7b). Moreover, spheroid assays of HLF-192KO cells were performed in different conditioned medium settings. The number of spheroids of HLF-192KO cells was significantly lower under exposure to conditioned medium from co-culture of HLF-192KO with LX2^si-*NDRG3*^ or LX2^si-*MCT1*^ than from co-culture of HLF-192KO with LX2^si-Ctrl^ (Fig. 7c). CD44^+^ and CD24^+^ HLF-192KO populations were also significantly reduced when they were co-cultured with LX2^si-*NDRG3*^ or LX2^si-*MCT1*^ (Fig. 7d, Supplementary Fig. S8c). As a control, HLF-192KO with overexpressed miR-192-5p did not exhibit significant alteration of migration, spheroid formation and CSC populations when co-cultured with different LX2 cells (Fig. 7b-d). Consistent data were obtained using a second set of siRNAs for *NDRG3* and *MCT1* (Supplementary Fig. S9). Therefore, blocking the lactate/ERK pathway in HCC microenvironmental cells suppressed the malignancy and stemness features of HCC cells in co-culture experiments.

In both HCC cohorts, patients were divided into four groups based on miR-192-5p expression in their tumor tissues (HCC^192Low^ and HCC^192High^, medium cut-off) and levels of NDRG3 and MCT1 in non-tumor tissues (NT^High_NDRG3 or MCT1^ and NT ^Low_NDRG3 and MCT1^, medium cut-offs). There was no expression difference of NDRG3 or MCT1 in non-tumor tissues between HCC^192Low^ and HCC^192High^ patients (Fig. 7e, Supplementary Fig. S10a). In Cohort 1, patients with HCC^192High^ NT^Low_NDRG3 and MCT1^ had the best prognosis, as shown by a prolonged time to recurrence and overall survival. In HCC^192Low^ subgroup, patients with NT^High_NDRG3 or MCT1^ had worse prognosis compared to patients with NT^Low_NDRG3 or MCT1^ (Fig. 7f). Similar but less significant data were obtained in Cohort 2, which might be due to the limited number of patients (*n* = 49) with available non-tumor mRNA data (Supplementary Fig. S10b). GSEA analysis in HCC^192Low^ patients revealed that several stem cell related gene-sets were enriched in patients with NT^High_NDRG3 or MCT1^ (Supplementary Fig. S10c-d). Together, HCC cells with miR-192-5p loss exhibited a highly malignant feature when they were surrounded by environmental non-tumors with high MCT1 or NDRG3 expression.

## Discussion

HCC is one of the most malignant cancers worldwide. Although a trend of decreasing incidence has been observed for many cancers, the incidence of HCC is still rising in both developing and developed countries [44]. CSCs are thought to be responsible for tumorigenesis as well as tumor metastasis and targeting CSCs holds the hope of eliminating cancer [3, 4, 6]. In the last decade, researchers’ attention has been drawn to the field of hepatic CSCs, leading to the identification of multiple hepatic CSC biomarkers. Accordingly, accumulated studies have revealed gene signatures and regulatory signaling pathways of these identified hepatic CSCs [18]. In this study, we aimed to understand essential metabolic features of hepatic CSCs.

MiR-192-5p, a liver-abundant miRNA, is functionally important in suppressing the stemness and malignancy features of HCC cells, and its genetic silencing frequently occurs in multiple groups of CSC^+^ HCCs, i.e., EpCAM^+^, CD90^+^, CD133^+^, CD44^+^, and CD24^+^ HCCs [23]. Here, we revealed that the miR-192-5p loss drove HCC cells to a hyperglycolytic metabolism status via targeting c-Myc and two glycolytic enzymes GLUT1 and PFKFB3, and a c-Myc/miR-192-5p positive feedback pathway. Five groups of CSC^+^HCCs presented a hyperglycolytic signature, high level of c-Myc and low miR-192-5p. Mechanistically, loss of miR-192-5p led to increased expression of its target genes, i.e., PFKFB3, GLUT1, and c-Myc, which facilitated a Warburg effect favored by cellular growth and stemness. Moreover, c-Myc also directly suppressed miR-192-5p expression, ensuring a positive feedback to drive hyperglycolytic features in CSC^+^HCCs. In addition, targeting PFKFB3, GLUT1, and c-Myc could also reduce hepatic CSC populations. Thus, hyperglycolysis was a key feature for CSC^+^ HCCs and effective methods of targeting hyperglycolytic cells in combination with conventional anticancer methods might effectively eliminate CSCs, leading to a significant reduction of HCC recurrence and metastasis. However, it remains unclear whether liver cells gain their CSC features and hyperglycolysis features at the same time or if one is the consequence of the other. The efforts to answer these questions will further improve our understanding of HCC initiation and may open new avenues to prevent HCC tumor initiation or metastasis.

The HCC microenvironment is complex, including but not limited to activated hepatic stellate cells, Kupffer cells, and hypoxia etc., and is believed to actively interact with HCC tumors as well as affect their malignancy status [7, 45, 46]. In our study, LX2 and THP1 (representing hepatic stellate cells and macrophages respectively) were used to co-culture with HCC cells in vitro. We found that hyperglycolytic HCC cells with miR-192-5p loss nourished LX2 and THP1 cells with overproduced lactate, which stimulated the pERK pathway in LX2 and THP1 cells via NDRG3/MCT1. Consistently, in the in vitro co-culture system, LX2 and THP1 cells with high levels of NDRG3 and/or MCT1 facilitated a more malignant phenotype of HCC cells with miR-192-5p loss. In vivo, HCC patients with low level of miR-192-5p in their tumor and high level of *NDRG3/MCT1* in their non-tumor had the shorter overall survival when compared to other patient subgroups. Thus, hepatic CSC-related metabolism affected the CSC microenvironment via accumulated lactate, and lactate in turn interacted with environmental cellular components (LX2 and THP1) that contributed to HCC stemness features. This was consistent with reported studies demonstrating that the structural and cellular components of the CSC microenvironment played important roles in CSC plasticity, proliferation and invasion [8, 9]. It remains undiscovered in our study how the pERK pathway in THP1 or LX2 cells promote HCC malignancy. It would be interesting to perform an in-depth study on cytokines or exosomes being produced by THP1 and LX2 cells through the lactate/ERK axis, and to explore their roles in promoting the malignancy of tumor cells. Both lactate and LX2/THP1 cells in the hepatic CSC environment contributed to the stemness and malignant features of HCC cells with miR-192-5p loss. Thus, we would need more insight on the interaction of tumor initiating cells and their environment to offer a better solution of cancer therapy regarding CSC elimination. Investigating the CSC-niche metabolism would be also important for the further development of metabolic targeting approaches, which might significantly impact cancer therapy.

As a class of non-coding RNAs, miRNAs participate in many key events in hepatocarcinogenesis. As a liver-specific and abundant miRNA, miR-192-5p is significantly down-regulated in HCC tumor tissues due to a high frequency of *TP53* mutation and its promoter hypermethylation [23]. In HCC cells, miR-192-5p loss not only led to increased populations of hepatic CSCs [23] and cell malignancy phenotype [23–25], but also a hyperglycolytic phenotype as revealed in this study (Fig. 7g). These findings suggested a potentially important role of miR-192-5p in regulating hepatic carcinogenesis. It will be essential to establish conditional miR-192-5p knockout mice and further investigate whether and at which stages miR-192-5p regulates hepatic carcinogenesis, the metabolism of HCC tumor and tumor microenvironment, as well as roles of the miR-192-5p-related metabolic pathways in regulating hepatic carcinogenesis in the mouse model.

## Conclusions

Taken together, loss of miR-192-5p promoted hyperglycolysis and stemness features in CSC^+^HCC cells through upregulating c-Myc and two glycolytic enzymes (PFKFB3 and GLUT1), and through a miR-192-5p/c-Myc positive feedback circuit. Moreover, HCC cells with miR-192-5p loss could also actively interact with their environmental non-tumor cells to produce a more aggressive and stemness-related feature via the over-produced lactic acid by HCC cells and consequently activating a lactate/MCT1/NDRG3/pERK pathway in their environmental non-tumor cells (Fig. 7g).

## Supplementary Information

**Additional file 1: Table S1.** Primers and oligos used in this study. **Table S2.** Antibodies used in this study. **Table S3.** Summary of metabolites significantly correlated with miR-192-5p. **Figure S1.** Glycolysis-related metabolites and genes in HCC subgroups with different levels of miR-192-5p or CSC biomarkers. **Figure.S2.** MiR-192-5p suppressed glycolysis-related genes and miR-192-5p knockout HCC cells presented the increased CSC features. **Figure S3.** The expression of cancer stem cell biomarkers and glycolytic enzymes, as well as lactate production in four additional 192KO clones. **Figure S4.** miR-192-5p KO cells consumed more glucose from the environment. **Figure S5.** miR-192-5p targets in regulating CSC and glycolytic features of HCC cells. **Figure S6.** Expression of p53 protein in HCC cells after exposure to Nutlin-3a, and hierarchical clustering of 76 c-Myc target genes. **Figure S7.** In CSC^+^ and CSC^−^ HCCs, expression levels of glycolytic genes and miR-192-5p as well as percentages of cases with c-Myc activation status defined in Fig. S6. **Figure S8.** The expression comparison of *MCT1* and *MCT4* in two HCC cohorts and phosphorylation of ERK in LX2, THP1 and HL7702 cells in co-culture with HLE HCC cells. **Figure S9.** Silencing *MCT1* and *NDRG3* in LX2 or THP1 cells reduced malignancy and stemness features of co-cultured 192KO HCC cells. **Figure S10.** The expression of *MCT1* or *NDRG3* in non-tumor from HCC patients and GSEA analysis in HCC^192Low^ patients using genes differentially expressed between cases with high levels of *MCT1* or *NDRG3* in their non-tumors and ones with low levels of *MCT1* and *NDRG3* in their non-tumors.

## Data Availability

miRNA transcriptome in HCC Cohort 1, GSE6857; mRNA transcriptome in HCC Cohort 1, GSE14520; metabolomics data in HCC Cohort 1 were referenced in Ref 12. miRNA sequencing and mRNA sequencing data were downloaded from TCGA. TP53 mutation status and MYC amplification information in HCC Cohort 2 were obtained from www.cbioportal.org/index.do.

## Abbreviations

CSC: Cancer stem cell
DMEM: Dulbecco’s modified Eagle’s medium
ECAR: Extracellular acidification rate
F6P: Fructose-6-Phosphate
GC/TOF-MS: Gas chromatography/time-of-flight mass spectrometry
GSEA: Gene Set Enrichment Analysis
G6P: Glucose-6-Phosphate
HCC: Hepatocellular carcinoma
HSC: Hepatic stellate cell
JCRB: Japanese Collection of Research Biosources Cell Bank
KEGG: Kyoto Encyclopedia of Genes and Genomes
OCR: Oxygen consumption rate
qRT-PCR: Quantitative reverse transcription polymerase chain reaction
sgRNAs: Single-guide RNAs
NADPH: Nicotinamide Adenine Dinucleotide Phosphate
TCGA: The Cancer Genome Atlas
2-DG: 2-deoxyglucose
2-NBDG: 2-(N-(7-nitrobenz-2-oxa-1,3-diazol-4-yl)amino)-2-deoxy-D-glucose
