# Supplementary material for: Loss of miR-192-5p initiates a hyperglycolysis and stemness positive feedback in hepatocellular carcinoma
Source: J Exp Clin Cancer Res. 2020 Nov 30;39:268. doi: 10.1186/s13046-020-01785-7 (PMC7708108; doi:10.1186/s13046-020-01785-7)

**Loss of MiR-192-5p Initiates a Hyperglycolysis and Stemness Positive Feedback in Hepatocellular Carcinoma.**

**Supplementary Tables**

**Table S1.** Primers and oligos used in this study.

**Table S2.** Antibodies used in this study.

**Table S3.** Summary of metabolites significantly correlated with miR-192-5p.

**Supplementary Figures**

**Fig. S1.** Glycolysis-related metabolites and genes in HCC subgroups with different levels of miR-192-5p or CSC biomarkers.

**Fig. S2.** MiR-192-5p suppressed glycolysis-related genes and miR-192-5p knockout HCC cells presented the increased CSC features.

**Fig. S3**. The expression of cancer stem cell biomarkers and glycolytic enzymes, as well as lactate production in four additional 192KO clones.

**Fig. S4.** miR-192-5p KO cells consumed more glucose from the environment.

**Fig. S5.** miR-192-5p targets in regulating CSC and glycolytic features of HCC cells.

**Fig. S6.** Expression of p53 protein in HCC cells after exposure to Nutlin-3a, and hierarchical clustering of 76 c-Myc target genes.

**Fig. S7.** In CSC^+^ and CSC^-^ HCCs, expression levels of glycolytic genes and miR-192-5p as well as percentages of cases with c-Myc activation status defined in Fig. S6.

**Fig. S8.** The expression comparison of *MCT1* and *MCT4* in two HCC cohorts and phosphorylation of ERK in LX2, THP1 and HL7702 cells in co-culture with HLE HCC cells.

**Fig. S9.** Silencing *MCT1* and *NDRG3* in LX2 or THP1 cells reduced malignancy and stemness features of co-cultured 192KO HCC cells.

**Fig. S10.** The expression of *MCT1* or *NDRG3* in non-tumor from HCC patients and GSEA analysis in HCC^192Low^ patients using genes differentially expressed between cases with high levels of *MCT1* or *NDRG3* in their non-tumors and ones with low levels of *MCT1* and *NDRG3* in their non-tumors.

**Table S1. Primers and Oligoes used in this study.**

| **Primers** | **Sequence** |
| --- | --- |
|  | **SYBR green real-time PCR** |
| *GLUT1* | F: 5’-TCACTGTGCTCCTGGTTCTG-3’ |
|  | R: 5’-CCTGTGCTCCTGAGAGATCC-3’ |
| *HK2* | F: 5’-TAGGGCTTTGAGAGCACCTGT-3’ |
|  | R: 5’-CCACACCACTGTCACTTTG-3’ |
| *PFKP* | F: 5’-CGCCTACCTCAACGTGGTG-3′ |
|  | R: 5’-ACCTCCAGAACGAAGGTCCTC-3′ |
| *PFKFB3* | F: 5’-ATTGCGGTTTTCGATGCCAC-3′ |
|  | R: 5’-GCCACAACTGTAGGGTCGT-3′ |
| *ALDOA* | F: 5’-ATGCCCTACCAATATCCAGCA-3′ |
|  | R: 5’-GCTCCCAGTGGACTCATCTG-3′ |
| *ENO2* | F: 5’-AGGTGCAGAGGTCTACCATAC-3′ |
|  | R: 5’-AGCTCCAAGGCTTCACTGTTC-3′ |
| *PKM2* | F: 5’-ATGTCGAAGCCCCATAGTGAA-3′ |
|  | R: 5’-TGGGTGGTGAATCAATGTCCA-3′ |
| *LDHA* | F: 5′-ACGTCAGCAAGAGGGAGAAA-3′ |
|  | R: 5′-CGCTTCCAATAACACGGTTT-3′ |
| *MCT4* | F: 5′-CCATGCTCTACGGGACAGG-3′ |
|  | R: 5′-GCTTGCTGAAGTAGCGGTT-3′ |
| *MCT1* | F: 5′-GGTGGAGGTCCTATCAGCAGT-3′ |
|  | R: 5′-CAGAAAGAAGCTGCAATCAAGC-3′ |
| *NDRG3* | F: 5′-GCAGCTTCCAAACTCTCTGG-3′ |
|  | R: 5′-TATAGGGTTCAGGCGGGAAT-3′ |
| *CYP1A2* | F: 5′-CTGGGCACTTCGACCCTTAC-3′ |
|  | R: 5′-TCTCATCGCTACTCTCAGGGA-3′ |
| 18s | F: 5′-GACTCAACACGGGAAACCTC-3′ |
|  | R: 5′-AGCATGCCAGAGTCTCGTTC-3′ |
|  | **For sgRNAs** |
| sgRNA-L | 5’-GCTGCCGAGACCGAGTGCAC-3’ |
| sgRNA-R | 5’-TAGGTCACAGGTATGTTCGC-3’ |
|  | **For mir-192 promoter region** |
| Region (-266,+186) | F: 5’-GGGGTACCTGGGGCAGCAGGCTGG-3’ *(_, KpnI site)* |
|  | R: 5’-CCGCTCGAGGAAGGGGCTCGTCCC-3’ *(_, XhoI site)* |
| Region (-367,+186) | F: 5’-GGGGTACCTGCTCTGTGGCCCCTTCT-3’ *(_, KpnI site)* |
|  | R: 5’-CCGCTCGAGGCTGATGCTAAGGCCCCCTTAT-3’*(_, XhoI site)* |
| Region (-1330,-300) | F: 5’-GGGGTACCACTTTTCCTCCGGGGAGAAA-3’ *(_, KpnI site)* |
|  | R: 5’-CCGCTCGAGCGGGACAAAGCTTCTGCTCT-3’ *(_, XhoI site)* |
| Region (-1330,+233) | F: 5’-GGGGTACCACTTTTCCTCCGGGGAGAAA-3’ *(_, KpnI site)* |
|  | R: 5’-CCGCTCGAGGCTGATGCTAAGGCCCCCTTAT-3’ *(_, XhoI site)* |
|  | **SiRNA sequences** |
| Si-*GLUT1* | Sense: 5’-GGAAUUCAAUGCUGAUGAUGA-3’ |
|  | Antisense: 5’-UCAUCAUCAGCAUUGAAUUCC-3’ |
| Si-*GLUT1#2* | Sense: 5’-GUGCCAUACUCAUGACCAU-3’ |
|  | Antisense: 5’-AUGGUCAUGAGUAUGGCAC-3’ |
| Si-*PFKFB3* | Sense: 5’-GCCGCAUCGUGUACUACCUGAUGAA-3’ |
|  | Antisense: 5’-UUCAUCAGGUAGUACACGAUGCGGC-3’ |
| Si-*PFKFB3#2* | Sense: 5’-GCUGUGAAGCAGUACAGCUCCUAC-3’ |
|  | Antisense: 5’-GUAGGAGCUGUACUGCUUCACAGC-3’ |
| Si-*MYC* | Sense: 5’-GCUUCACCAACAGGAACUA-3’ |
|  | Antisense: 5’-UAGUUCCUGUUGGUGAAGC-3’ |
| Si-*MYC*#2 | Sense: 5’-CGUUAGCUUCACCAACA-3’ |
|  | Antisense: 5’-UGUUGGUGAAGCUAACG-3’ |
| Si-*NDRG3* | Sense: 5’-AGAUCAAACCACUUCUAAAUGAUAA-3’ |
|  | Antisense: 5’-UUAUCAUUUAGAAGUGGUUUGAUCU-3’ |
| Si-*NDRG3 #2* | Sense: 5’-AGUCAGAUGGAACUCAAGAAUCCUG-3’ |
|  | Antisense: 5’-CAGGAUUCUUGAGUUCCAUCUGACU-3’ |
| Si-*MCT1* | Sense: 5’-AUUUGGAAAAGUCAGCCUCUU-3’ |
|  | Antisense: 5’-AAGAGGCUGACUUUUCCAAAU-3’ |
| Si-*MCT1 #2* | Sense: 5’-CACCACCAGCGAAGUGUCAUGGAUA-3’ |
|  | Antisense: 5’-UAUCCAUGACACUUCGCUGGUGGUG-3’ |
| Si-*TP53*-001 | Sense: 5’-GCAUGAACCGGAGGCCCAU-3’ |
|  | Antisense: 5’-AUGGGCCUCCGGUUCAUGC-3’ |
| Si-*TP53*-002 | Sense: 5’-GACUCCAGUGGUAAUCUAC-3’ |
|  | Antisense: 5’-GUAGAUUACCACUGGAGUC-3’ |

**Table S2. Antibodies used in this study.**

| **Antibodies** | **Source** | **Identifier** |
| --- | --- | --- |
| c-Myc (Y69) | Abcam | Cat# ab32072 |
| PFKFB3 (EPR12594) | Abcam | Cat# ab181861 |
| GLUT1 | Proteintech | Cat# 21829-1-AP |
| PKM2 | Abcam | Cat# ab150377 |
| LDHA | HuaAn Biotechnology Co.,Ltd. | Cat# ER00702 |
| Aldoa (JM54-19) | HuaAn Biotechnology Co.,Ltd. | Cat# ET1705-91 |
| HK2 (F1-D3) | HuaAn Biotechnology Co.,Ltd. | Cat# EM1710-04 |
| NDRG3 | Abcam | Cat# ab131266 |
| MCT1(H-1) | Santa Cruz Biotechnology | Cat# sc-365501 |
| MCT4(D-1) | Santa Cruz Biotechnology | Cat# sc-376140 |
| PFKP (JU53-31) | HuaAn Biotechnology Co.,Ltd. | Cat# ET7106-97 |
| ERK1/2(137F5) | Cell Signaling Technology | Cat# 4695 |
| p-ERK1/2(Thr202/Tyr204) | Cell Signaling Technology | Cat# 4370 |
| Actin | Sigma-Aldrich | Cat# A5441 |
| EpCAM-APC (Clone HEA-125) | Miltenyi Biotec | Cat# 130-113-822 |
| CD133-APC (Clone AC133) | Miltenyi Biotec | Cat# 130-113-668 |
| CD24-APC (Clone 32D12) | Miltenyi Biotec | Cat# 130-099-399 |
| CD90-APC (Clone DG3) | Miltenyi Biotec | Cat# 130-097-935 |
| CD44-APC (Clone BJ18) | Biolegend | Cat# 338805 |

**Table S3. Summary of metabolites significantly correlated with miR-192-5p.**

| **Metabolites*** | **r-value** | **p-value** | **Related pathway** |
| --- | --- | --- | --- |
| **\|r-value\| > 0.5** | | | |
| NADPH | -0.78 | 0.04 | Glycolysis/Glycolysis side pathway |
| fructose-6-phosphate (F6P) | -0.51 | 0.02 | Glycolysis |
| glucose-6-phosphate (G6P) | -0.51 | 0.02 | Glycolysis |
| taurolithocholate 3-sulfate | 0.68 | 0.003 | Primary bile acid biosynthesis |
| hippurate | 0.63 | 0.05 | Amino acids biosynthesis |
| riboflavin (Vitamin B2) | 0.54 | 0.01 | Riboflavin metabolism |
| phosphoethanolamine | 0.53 | 0.01 | Phospholipid synthesis |
| **0.4< \|r-value\| < 0.5** | | | |
| galacturonate | -0.48 | 0.03 | Carbon metabolism |
| adenine | -0.47 | 0.05 | Purine metabolism |
| AMP | -0.47 | 0.03 | Phosphonate and phosphinate metabolism/ Purine metabolism |
| scyllo-inositol | -0.46 | 0.03 | Inositol phosphate metabolism |
| erythritol | -0.46 | 0.04 | Carbon metabolism/ ABC transporters |
| butyrylcarnitine | -0.45 | 0.04 | Fatty acid degradation |
| pipecolate | -0.45 | 0.04 | Tyrosine metabolism/ Amino acids biosynthesis |
| glycylleucine | -0.47 | 0.04 | Amino acids biosynthesis |
| serine | 0.47 | 0.03 | Glycolysis side pathway |
| glycine | 0.44 | 0.04 | Glycolysis side pathway |

^#^|r-values|>0.4, p-value<0.05

*****NADPH, nicotinamide adenine dinucleotide phosphate; AMP, adenosine 5'-monophosphate

**Supplementary Figures**

**Fig. S1. Glycolysis-related metabolites and genes in HCC subgroups with different levels of miR-192-5p or CSC biomarkers.**

(a) Expression levels of G6P, F6P and NADPH in tumors and non-tumors from HCC patients with miR-192-5p low and high levels in tumors. The medium cut-off of miR-192-5p was used. (b) Glycolysis-related genes expression in non-tumor from HCC patients with miR-192-5p low and high levels in tumors. (c,d) For each CSC biomarker, patients were divided to CSC^+^ and CSC^-^ HCC cases based on the quartile level of CSC biomarker. Glycolysis-related genes expression in tumor (c) and non-tumor (d) were compared between CSC^+^ HCCs and CSC^-^ HCCs. (e) In Cohort 2, levels of glycolysis-related genes were compared in tumor between patients with high and low mir-192 expression, and between CSC^+^ HCCs and CSC^-^ HCCs. (b,c) Student t-test was used. (a,d,e) Non–parametric t-test was used. (***, p<0.001; **, p<0.01; *, p<0.05).

**Fig. S2**. **miR-192-5p suppressed glycolysis-related genes and miR-192-5p knockout HCC cells presented the increased CSC feature.**

(a) Luciferase activities of Zip192 reporter in Huh7 cells infected with miRZip-ctrl or miRZip-192. miR-192-5p level in HLF and HLE cells infected with lentivirus pmiR-ctrl or pmiR-192. (b) RT-qPCR analysis of glycolysis-related genes in Huh7 cells infected with lentivirus miRZip-ctrl or miRZip-192, and in HLF and HLE cells with overexpressed miR-192-5p. (c) mir-192 fragment examination by PCR in an wild-type (WT) clone and miR-192-5p knockout (192KO) clones of HLF HCC cells. (d) mir-192 fragment examination by PCR in WT clone and 192KO clones of HLE cells. (e) Flow cytometry analysis using APC-conjugated antibodies against different CSC biomarkers in 192KO lines and WT lines of HLF and HLE cells. (f) RT-qPCR analysis of a group of CSC biomarkers in 192KO lines and WT lines of HLF and HLE cells. *CYP1A2* was measured as a negative control. (g) Spheroid formation assays in an ultra-low attachment plate were performed using HLF- 192KO cells and HLF-WT cells.

**Fig. S3**. **The expression of cancer stem cell biomarkers and glycolytic enzymes, as well as lactate production in four additional 192KO clones.**

(a). RT-qPCR analysis of glycolysis-related genes in two extra 192KO clones (192KO ex#1 and 192KO ex#2) and an extra WT clone (WT ex#1) derived from HLF and HLE HCC cell lines, respectively. (b) Western blot analysis of glycolytic enzymes in 192KO ex#1, 192KO ex#2 and WT ex#1 of HLF and HLE HCC cells, respectively. (c) Measurement of lactate in cultured medium at different time points in 192KO ex#1, 192KO ex#2 and WT ex#1 of HLF and HLE HCC cells, respectively. (d) Metabolites detected by non-targeted Metabolomics detection of WT and 192KO lines for HLF cells infected with lentivirus pmiR-Ctrl or pmiR-192 in both internal cells and culture medium. Student t-test was used. *, p<0.05.

**Fig. S4**. **miR-192-5p KO cells consumed more glucose from the environment.**

(a) Glucose concentration in culture medium from Huh7 cells infected with lentivirus miRZip-Ctrl or miRZip-192. (b) Cell viability in Huh7 cells infected with lentivirus miRZip-Ctrl or miRZip-192 when cells were exposed to different doses of 2-DG (Right). (c) Flow cytometry analysis of HCC cells infected with lentivirus RFP. (d) Flow cytometry analysis of 2-NBDG uptake for all cells in the co-culture system. HLF cells infected with lenti-virus pmiR-Ctrl (RFP) or pmiR-192 (RFP) were co-cultured with LX2, HL7702 and THP1. (e) Flow cytometry analysis showed glucose uptake in HLE-WT or -192KO cells using different doses of 2-NBDG (10, 25, 30, 50 and 75uM). (f) Flow cytometry analysis of 2-NBDG uptake for all cells in co-culture system. HLE cells infected with lenti-virus pmiR-Ctrl (RFP) or pmiR-192 (RFP) were co-cultured with LX2 or HL7702. Two-way ANOVA analysis was performed for (a, b) and student t-test was performed for (f). *, p<0.05.

**Fig. S5**. **miR-192-5p targets in regulating CSC and glycolytic features of HCC cells.**

(a) ECAR was measured in HLF-WT and HLF-192KO cells transfected with 50nM si-Ctrl, si-*PFKFB3 #2*, si-*GLUT1 #2*, and si-*MYC #2* respectively. (b,c) In HCC Cohorts 1 (b) and 2 (c), mRNA expression of *PFKPB3*, *GLUT1*, and *MYC* in tumor and non-tumor tissues of HCC^192high^ patients and HCC^192low^ patients. (d) Flow cytometry analysis using APC-conjugated antibodies against CSC biomarkers CD24 and CD44 in HLF-WT cells transfected with si-Ctrl, HLF-192KO cells transfected with si-Ctrl, and HLF-192KO cells transfected with si-*MYC*. (b and d) Student t-test was used. (c) Non–parametric t-test was used. *, p<0.05.

**Fig. S6.** **Expression of p53 protein in HCC cells after exposure to Nutlin-3a, and hierarchical clustering of 76 c-Myc target genes.**

(a) Western blotting analysis for p53, and RT-qPCR analysis for miR-192-5p in different HCC cell lines treated with Nutlin-3a (10uM). (b) Western blotting analysis for p53 and c-Myc, and RT-qPCR analysis for miR-192-5p in HepG2 with silenced p53 and over-expressed c-Myc. Student t-test was performed. *, p<0.05. (c) Hierarchical clustering of 76 c-Myc target genes in Cohort 1. Each row represents an individual gene, and each column represents an individual case. (d) Hierarchical clustering of 76 c-Myc target genes in Cohort 2. Each row represents an individual gene, and each column represents an individual case.

**Fig. S7. In CSC^+^ and CSC^-^ HCCs, expression levels of glycolytic genes and miR-192-5p as well as percentages of cases with c-Myc activation status defined in Fig. S6.**

(a) In Cohort 1, there are 176 HCC patients with available miRNA and mRNA data. CSC^+^ and CSC^-^ HCC cases were defined based on the quarter cut-off level of each CSC biomarker in HCC tumors. Heat map was generated using the median expression levels of glycolytic genes and miR-192-5p in each group. Percentages of cases with c-Myc activation status were also shown. (b) In Cohort 2, 240 HCC patients have available MYC amplification information as well as transcriptome data. CSC^+^ and CSC^-^ HCC cases were defined based on the quarter cut-off level of each CSC biomarker in HCC tumors. Heat map was generated using the median expression levels of glycolytic genes and mir-192 in each group. Percentages of cases with c-Myc activation status and numbers of cases with *MYC* amplification were also shown.

**Fig. S8. The expression comparison of *MCT1* and *MCT4* in two HCC cohorts and phosphorylation of ERK in LX2, THP1 and HL7702 cells in co-culture with HLE HCC cells.**

(a) The relative expression of *MCT1* vs. *MCT4* in tumors and non-tumors from HCC patients. Paired student t-test was performed for Cohort 1. Unpaired t-test was performed for Cohort 2. (b) Western blotting analysis for pERK in LX2, THP1 and HL7702 cells, when they were co-cultured with HLE-WT and -192KO cells infected with lentivirus pmiR-Ctrl or pmiR-192 in a co-culture chamber system. (c) Flow cytometry analysis of CD24^+^ and CD44^+^ populations in HLF-192KO cells or HLF-192KO infected with p-miR-192/GFP cells co-cultured with LX2 pre-transfected with si-*NDRG3* or si-*MCT1*.

**Fig. S9. Silencing *MCT1* and *NDRG3* in LX2 or THP1 cells reduced malignancy and stemness features of co-cultured 192KO HCC cells.**

(a) Western blot analysis of NDRG3, MCT1, pERK and total ERK in LX2 cells with different treatments. (b) Western blot analysis of NDRG3, MCT1, pERK and total ERK in THP1 cells with different treatments. (c, d) For wound healing assay, scratches were generated in a confluent monolayer HLF cells infected with pmiR-ctrl/RFP (c) or pmiR-192/RFP (d) which were co-cultured with LX2 or THP1 pre-transfected with si-*NDRG3* #2 or si-*MCT1* #2. (e,f) HLF-192KO cells or HLF-192KO infected with pmiR-ctrl/GFP (e) or pmiR-192/GFP (f) were used for spheroid formation assay. Conditioned medium was used for this assay and collected from corresponding HLF cells co-cultured with LX2 pre-transfected with si-*NDRG3* #2 or si-*MCT1* #2.

**Fig. S10.** **The expression of *MCT1* or *NDRG3* in non-tumor from HCC patients and GSEA analysis in HCC^192Low^ patients using genes differentially expressed between** **cases with high levels of *MCT1* or *NDRG3* in their non-tumors and ones with low levels of MCT1 and NDRG3 in their non-tumors.**

(a) In Cohort 2, the relative levels of *MCT1* and *NDRG3* in non-tumor samples from patients with different levels of mir-192 in their tumors are depicated. (b) Kaplan–Meier curves of overall survival and time to recurrence in Cohort 2 according to mir-192 level in tumors as well as *MCT1* or *NDRG3* levels in non-tumors. (c) In HCCs^192Low^ patients, class comparison was performed between patients with high levels of *MCT1* or *NDRG3* in their non-tumors and ones with low levels of *MCT1* and *NDRG3* in their non-tumors. (d) GSEA analysis was performed to identify functionally related “gene sets” with statistically significant enrichment, using genes differentially expressed between HCCs^192Low^ with high levels of *MCT1* or *NDRG3* in their non-tumors and ones with low levels of *MCT1* and *NDRG3* in their non-tumors.


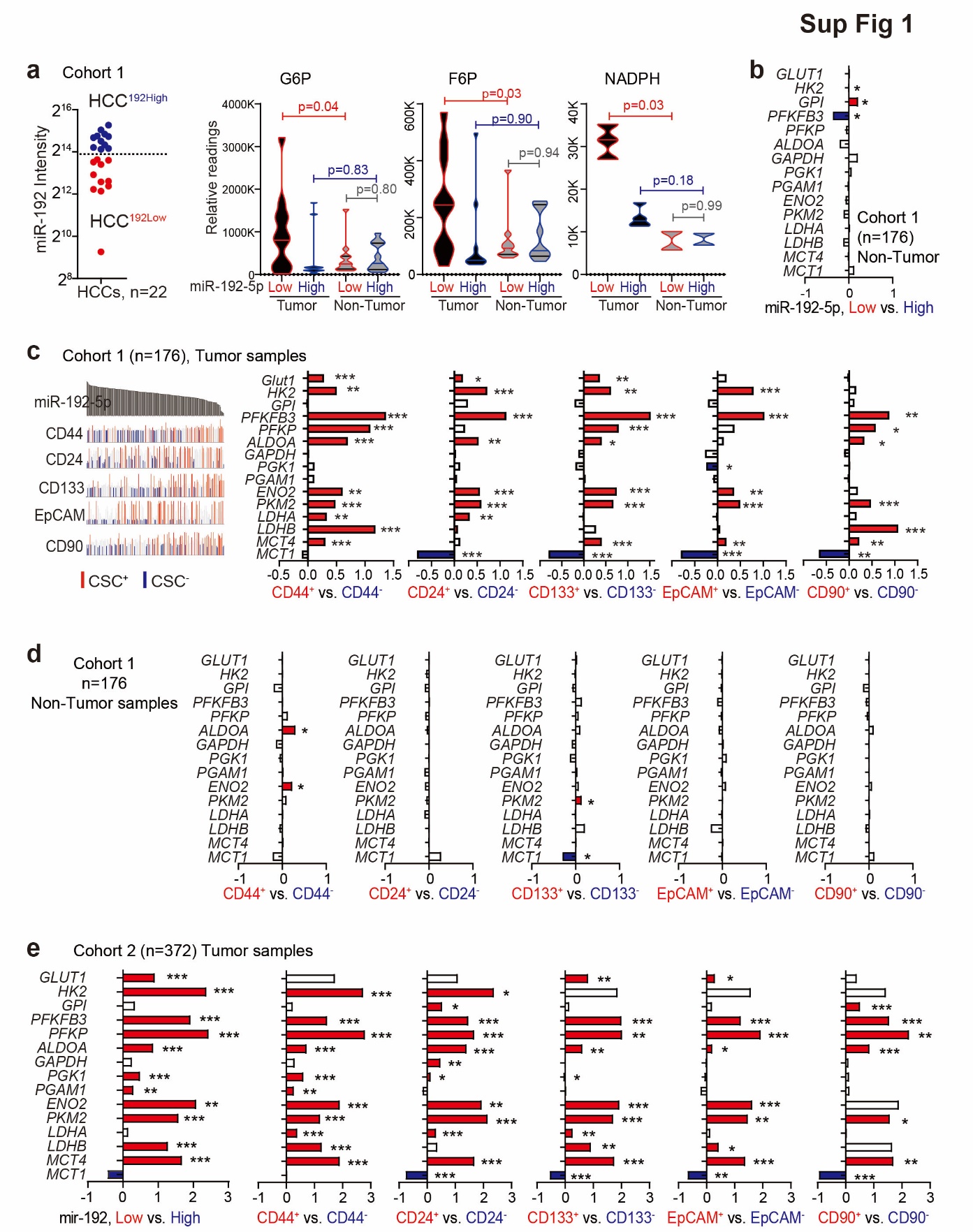


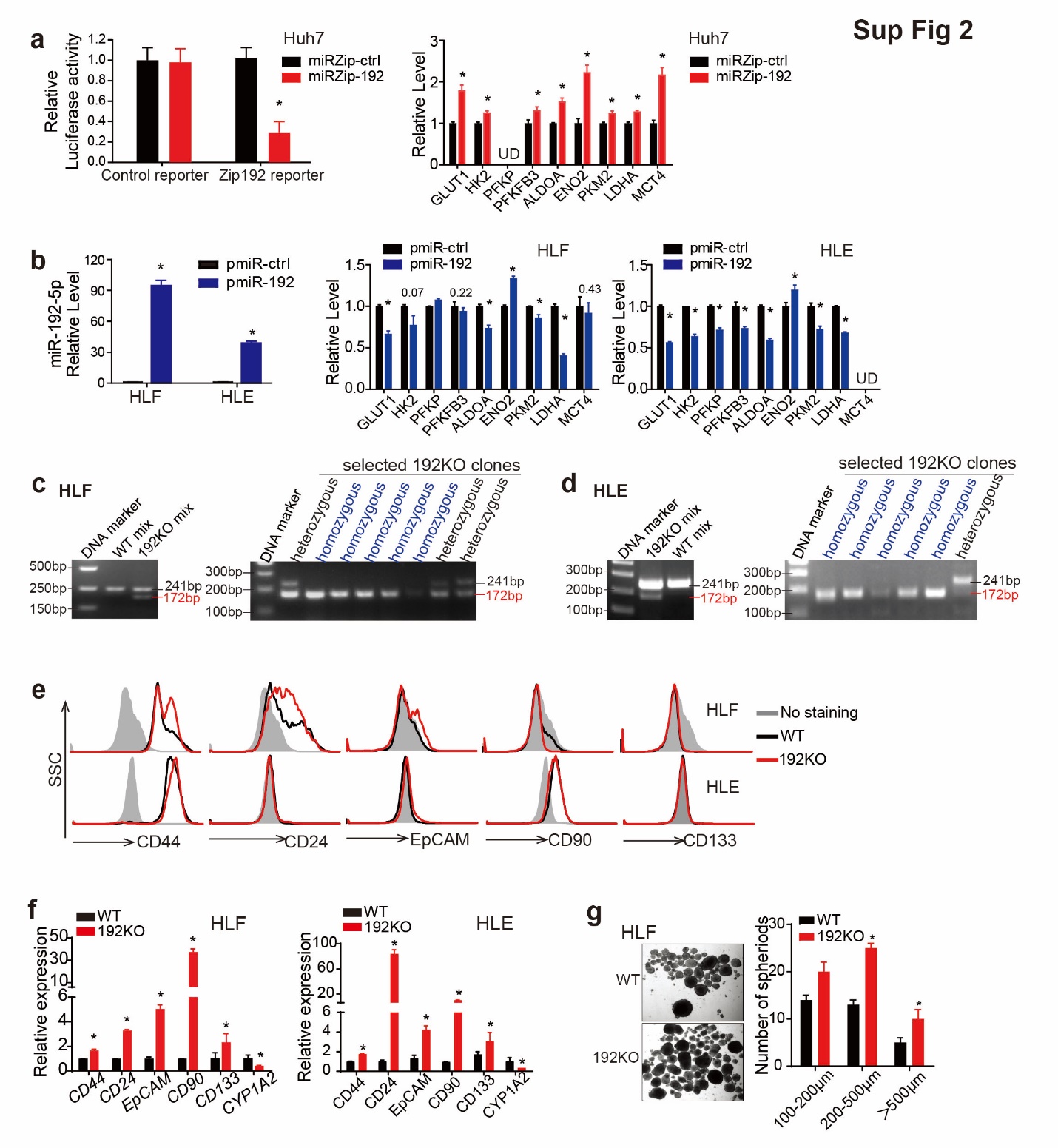


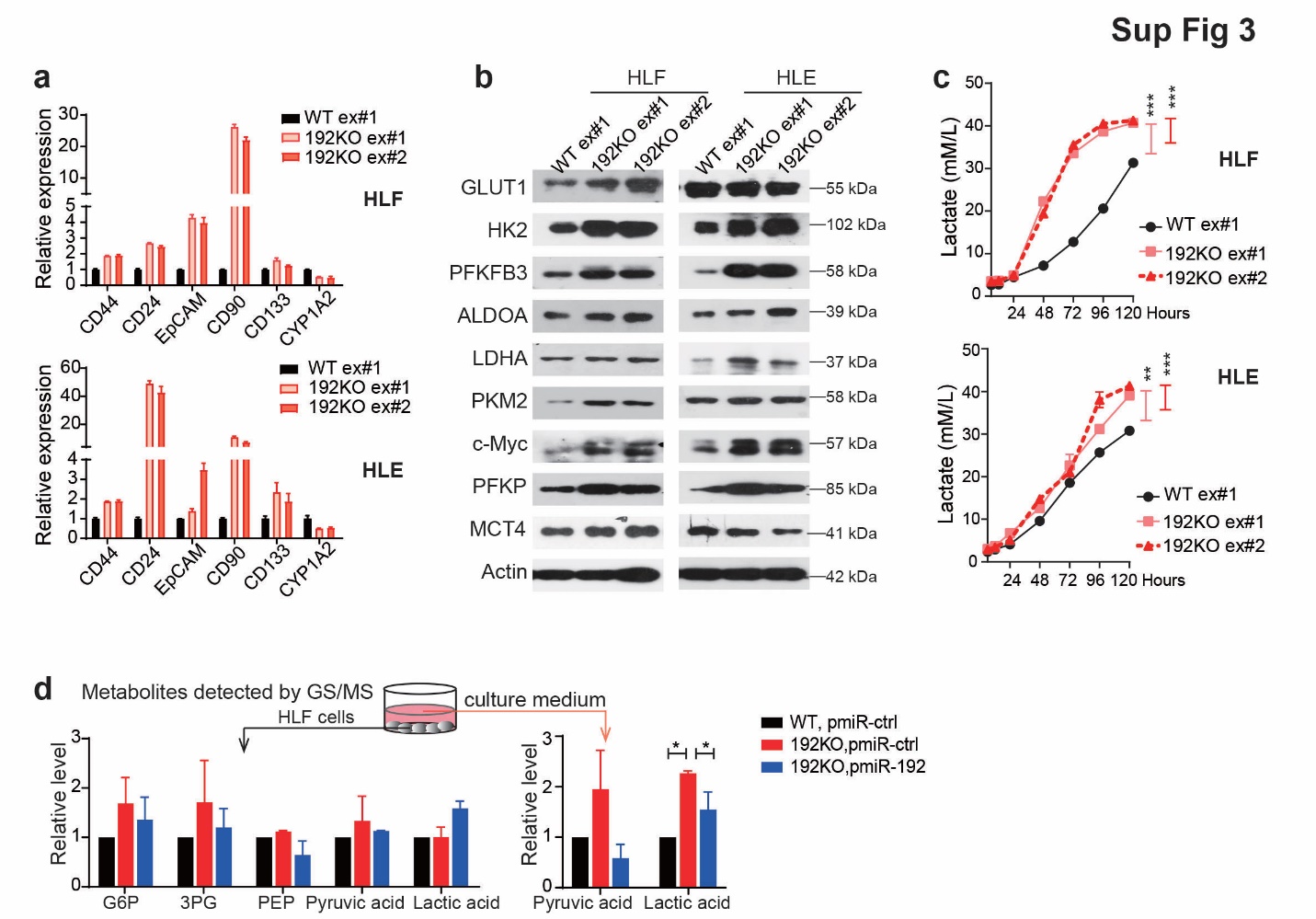


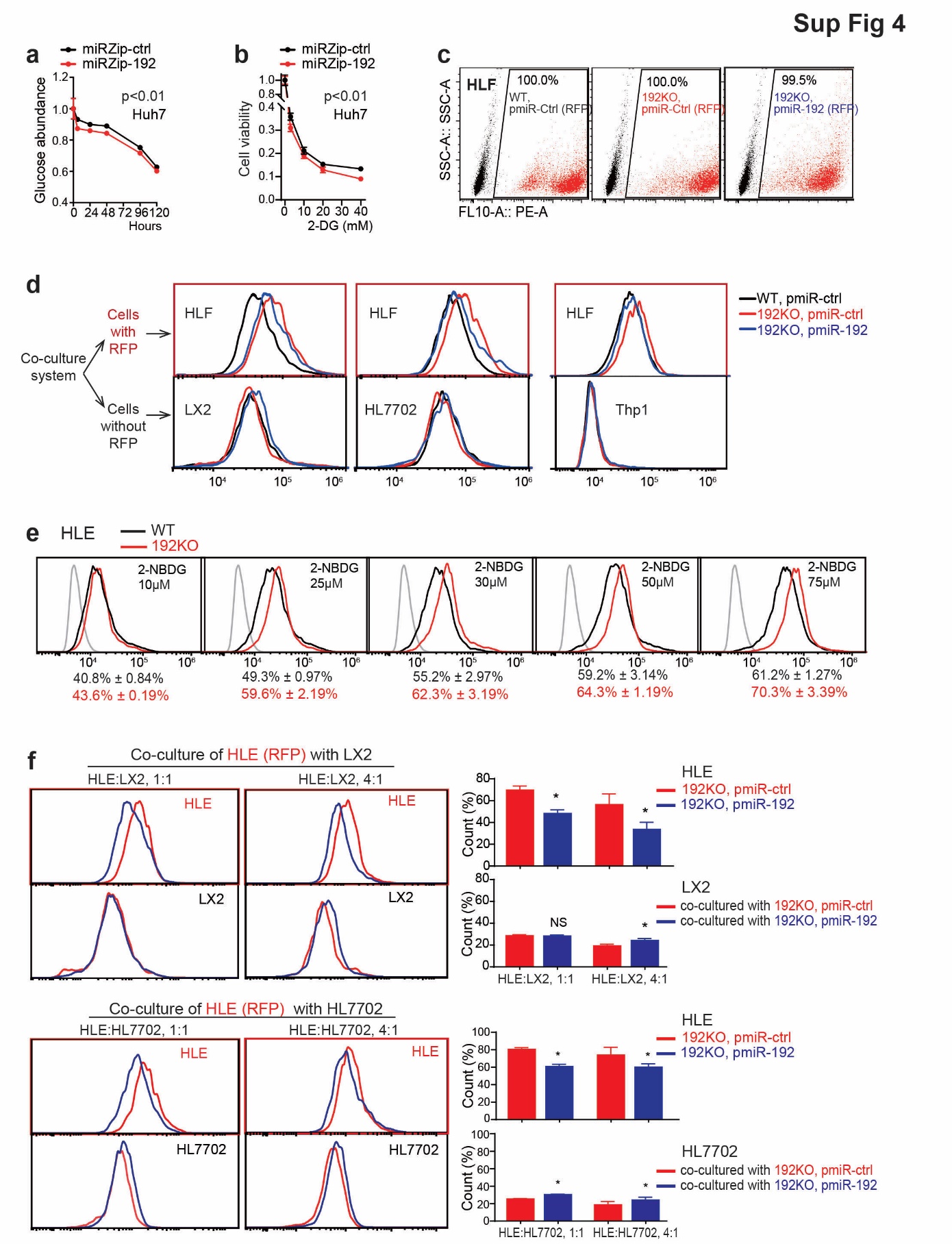


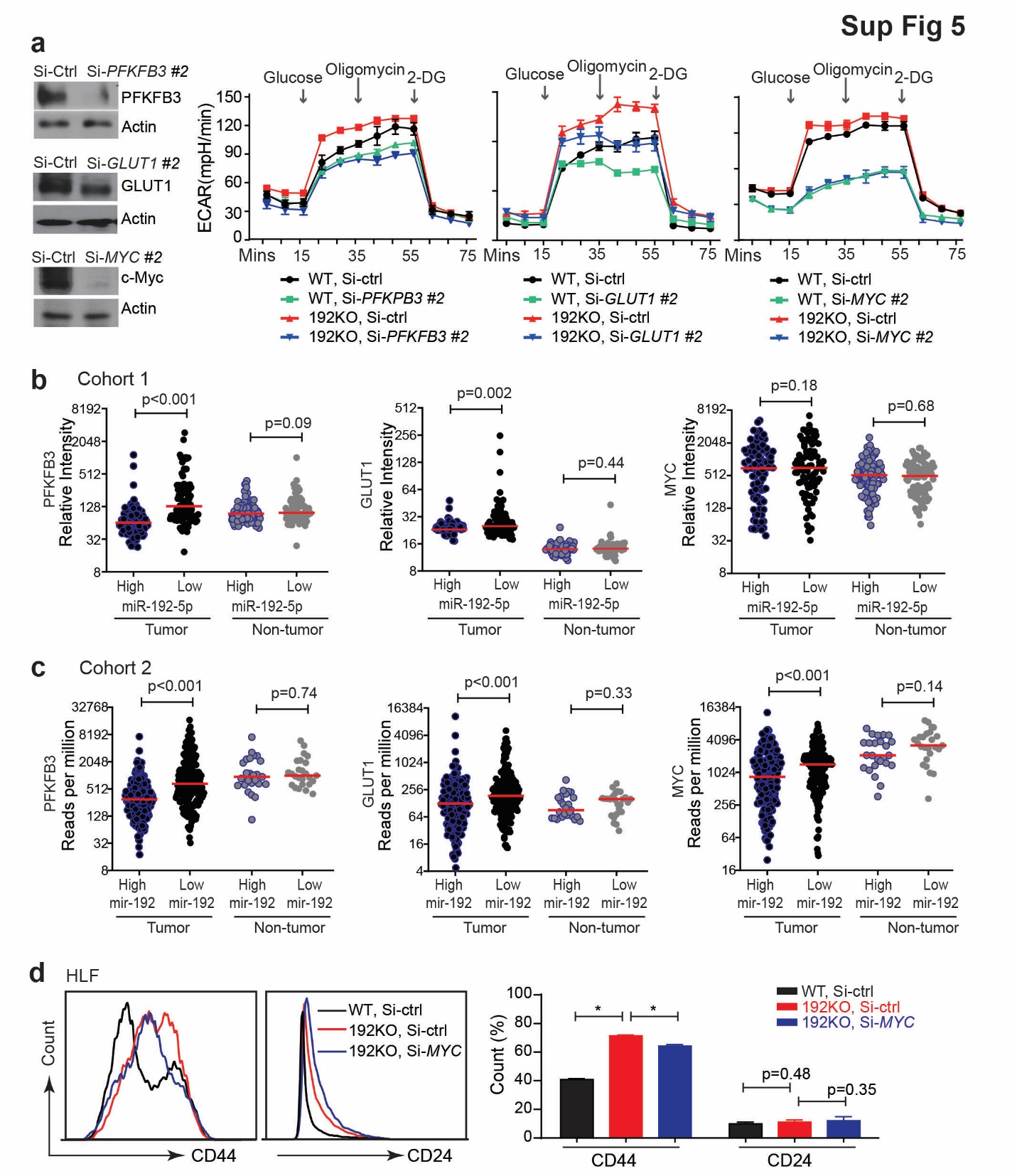


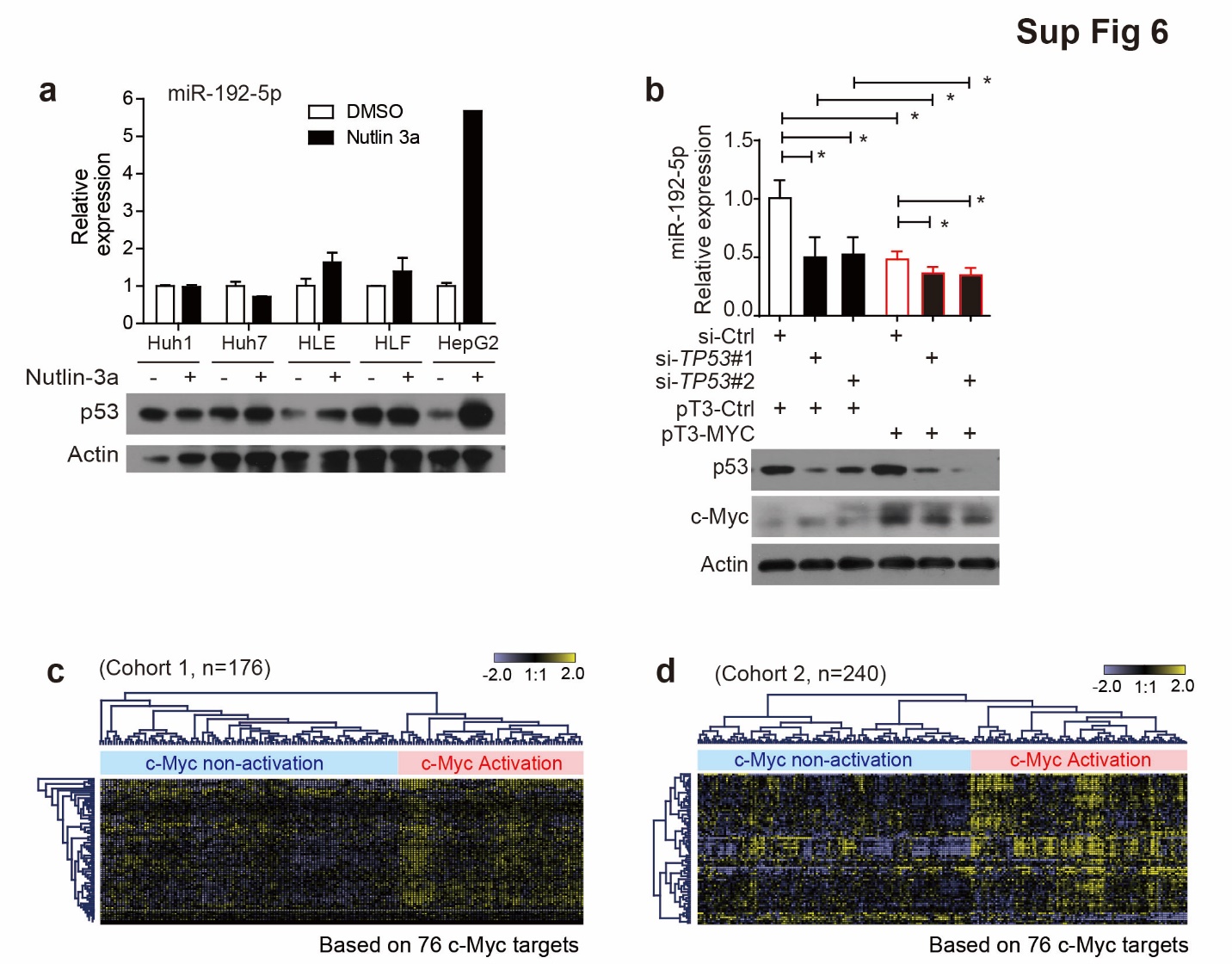


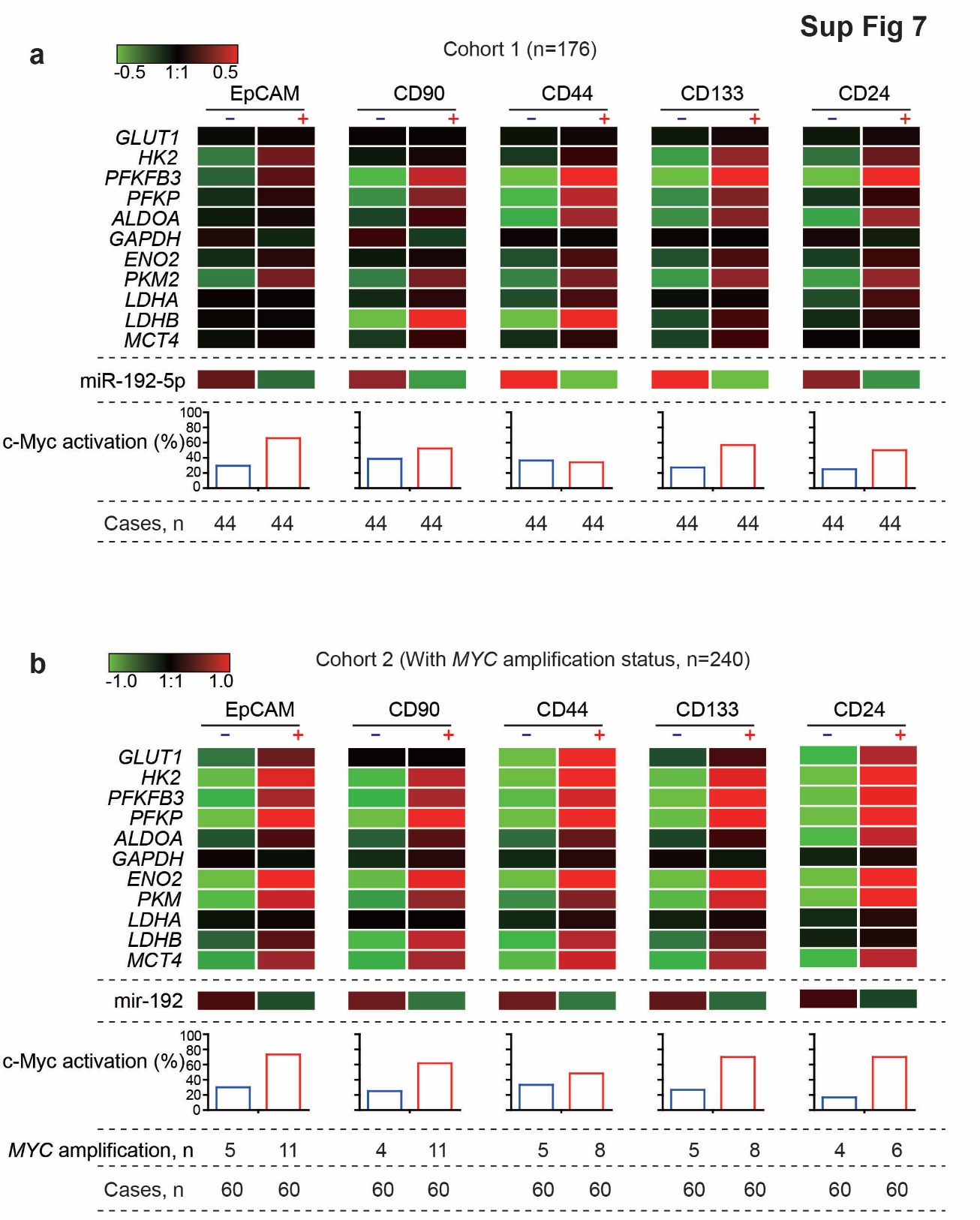


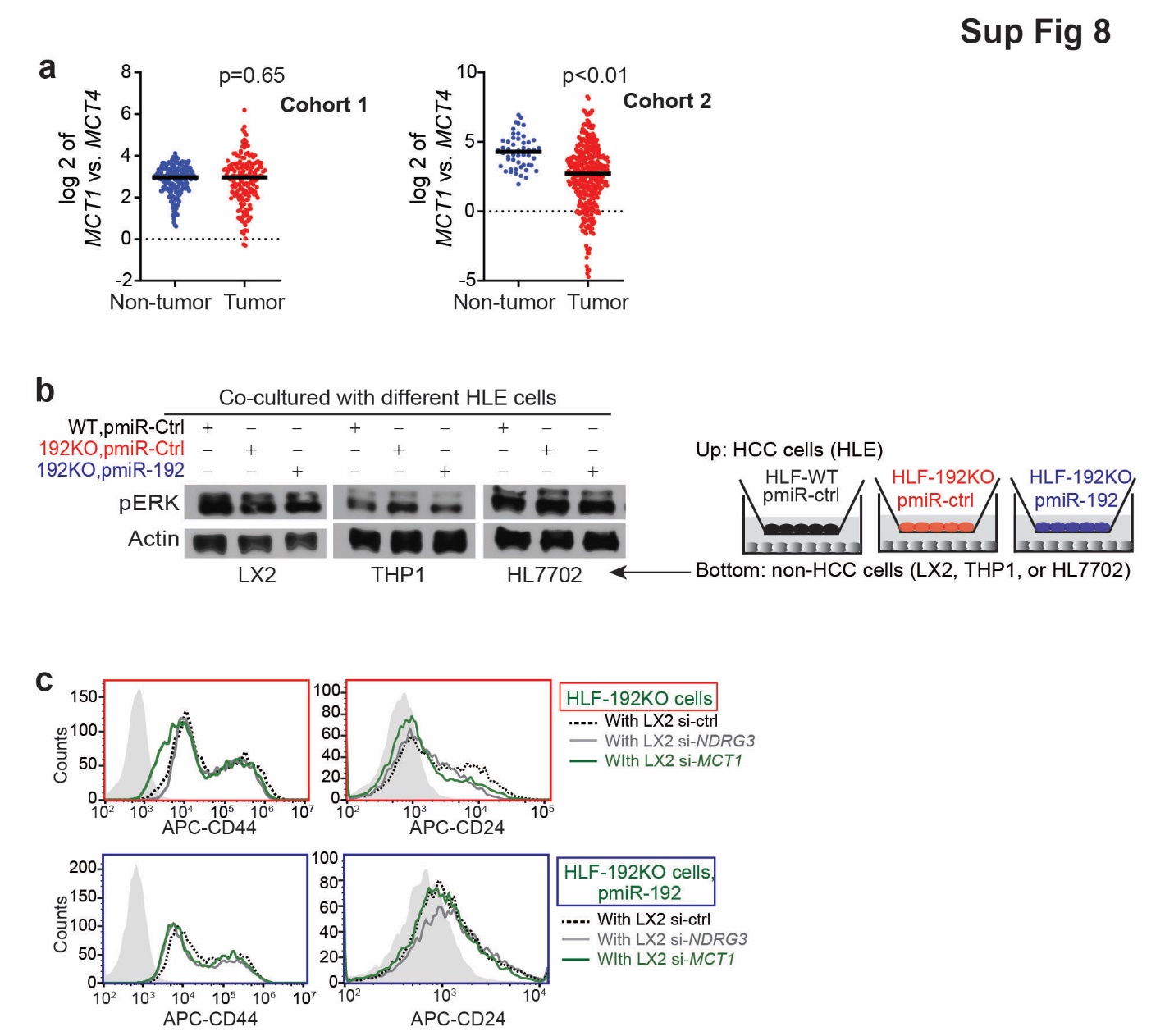


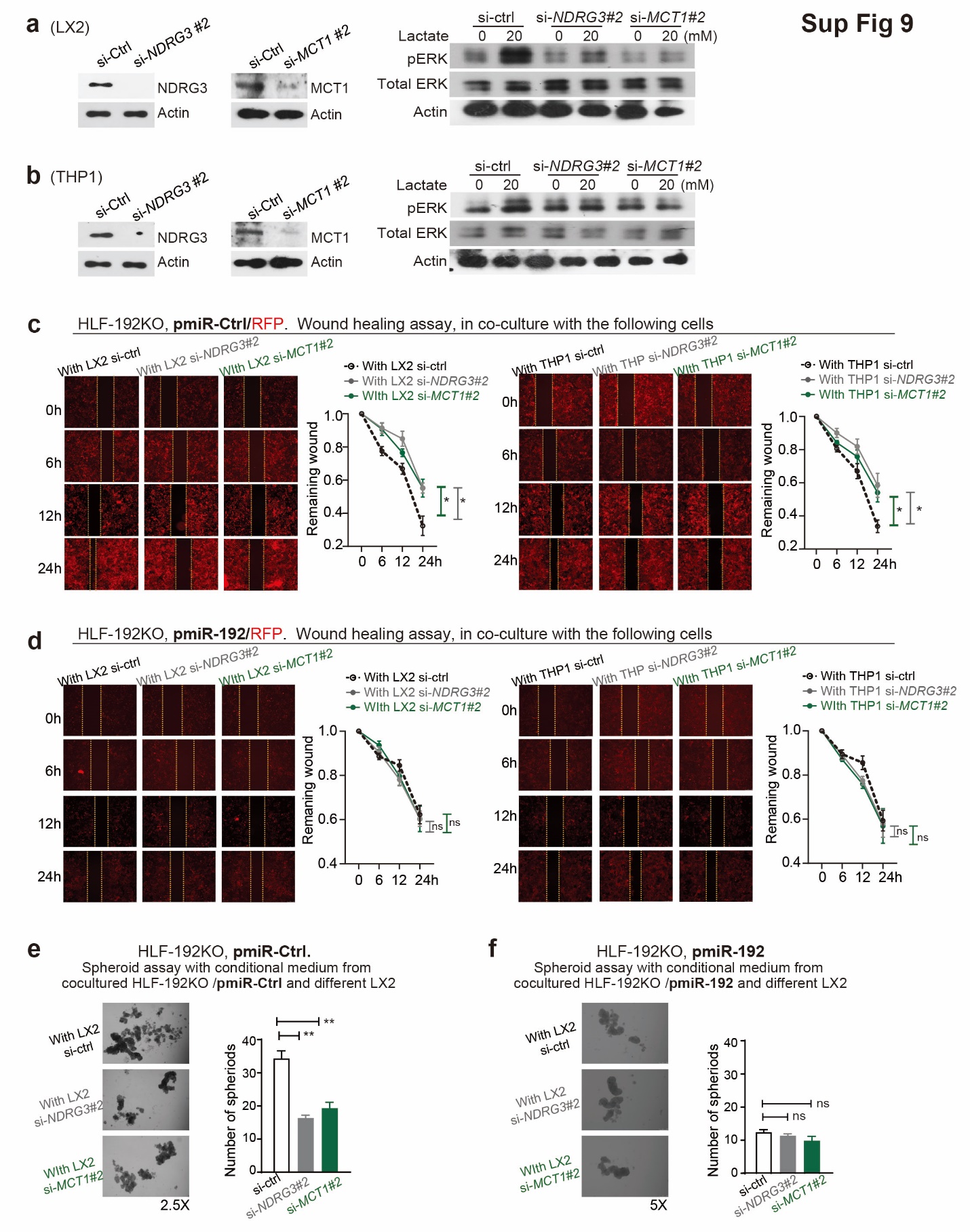


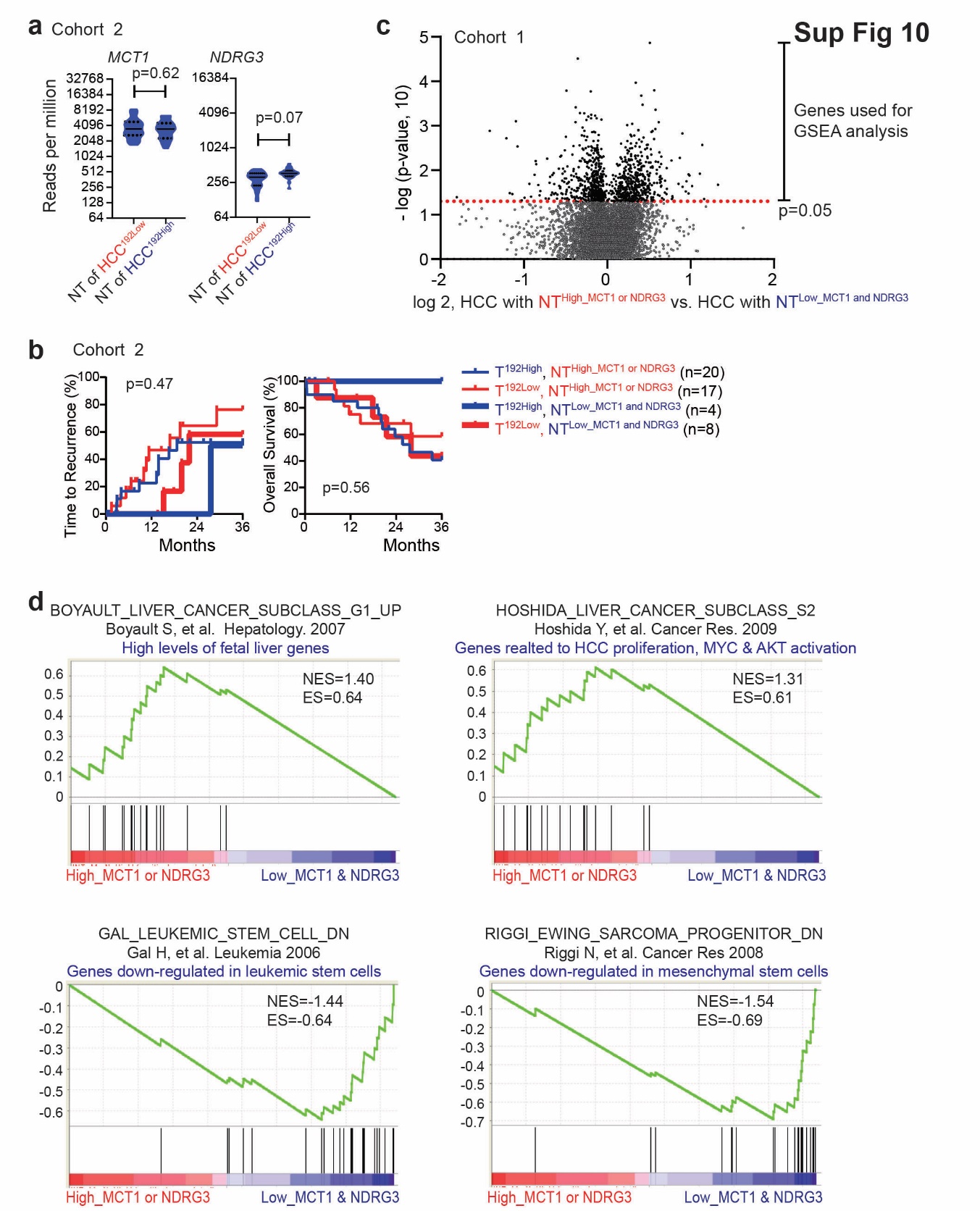

Supplement: Supplementary file 1 — Additional file 1: Table S1. Primers and oligos used in this study. Table S2. Antibodies used in this study. Table S3. Summary of metabolites significantly correlated with miR-192-5p. Figure S1. Glycolysis-related metabolites and genes in HCC subgroups with different levels of miR-192-5p or CSC biomarkers. Figure.S2. MiR-192-5p suppressed glycolysis-related genes and miR-192-5p knockout HCC cells presented the increased CSC features. Figure S3. The expression of cancer stem cell biomarkers and glycolytic enzymes, as well as lactate production in four additional 192KO clones. Figure S4. miR-192-5p KO cells consumed more glucose from the environment. Figure S5. miR-192-5p targets in regulating CSC and glycolytic features of HCC cells. Figure S6. Expression of p53 protein in HCC cells after exposure to Nutlin-3a, and hierarchical clustering of 76 c-Myc target genes. Figure S7. In CSC+ and CSC− HCCs, expression levels of glycolytic genes and miR-192-5p as well as percentages of cases with c-Myc activation status defined in Fig. S6. Figure S8. The expression comparison of MCT1 and MCT4 in two HCC cohorts and phosphorylation of ERK in LX2, THP1 and HL7702 cells in co-culture with HLE HCC cells. Figure S9. Silencing MCT1 and NDRG3 in LX2 or THP1 cells reduced malignancy and stemness features of co-cultured 192KO HCC cells. Figure S10. The expression of MCT1 or NDRG3 in non-tumor from HCC patients and GSEA analysis in HCC192Low patients using genes differentially expressed between cases with high levels of MCT1 or NDRG3 in their non-tumors and ones with low levels of MCT1 and NDRG3 in their non-tumors. [file 13046_2020_1785_MOESM1_ESM.docx]
